# Leveraging window-pane analysis with environmental factor loadings of genotype-by-environment interaction to identify high-resolution weather-based variables associated with plant disease

**DOI:** 10.3389/fpls.2025.1637130

**Published:** 2025-09-11

**Authors:** Vinicius C. Garnica, Peter S. Ojiambo

**Affiliations:** Center for Integrated Fungal Research, Department of Entomology and Plant Pathology, North Carolina State University, Raleigh, NC, United States

**Keywords:** disease prediction, environmental covariates, factor analytic model, feature engineering, moving average, stability selection

## Abstract

Designing and identifying biologically meaningful weather-based predictors of plant disease is challenging due to the temporal variability of conducive conditions and interdependence of weather factors. Confounding effects of plant genotype further obscure true environmental signals within observed disease responses. To address these limitations, this study leveraged window-pane analysis with feature engineering and stability selection, to identify weather-based variables associated with latent environmental factors ( $\hat{\lambda }$ ) of a factor analytic model explaining genotype-by-environment (GEI) effects on disease severity in multi-environment trials. Using Stagonospora nodorum blotch of wheat as a case study and a two-stage feature engineering procedure, hourly weather data, i.e., air temperature (*T*), precipitation (*R*), and relative humidity (*RH*), were aggregated into 1,530 distinct time series, in the first stage feature engineering procedure. These series were correlated daily with $\hat{\lambda }$ throughout the second half of the wheat growing season. In the second stage procedure, significant daily weather variables were consolidated into optimal epidemiological periods relative to wheat anthesis, yielding 60, 19, and 28 second-level weather-based variables derived from the first ( ${\hat{\lambda }}_{1}$ ), second ( ${\hat{\lambda }}_{2}$ ), and third ( ${\hat{\lambda }}_{3}$ ) environmental factor loadings, respectively. Among the weather-based predictors identified, *fa1.41_18.TRH.13T16nRH.G80.daytime.sum_25* and *fa1.11_5.R.S.dawn.sum_10*, were positively associated with ${\hat{\lambda }}_{1}$ (i.e., the dominant environmental gradient underlying variation in SNB severity across environments) pre-anthesis, during a period of 24 and 7 consecutive days, respectively. In contrast, *fa1.22_16.TR.19T22nR.G0.2.dawn.sum_20* and *fa1.2_-12.RH.L35.daytime.sum_15* were negatively associated with ${\hat{\lambda }}_{1}$ at pre-anthesis and post-anthesis, respectively. Additional predictors derived from *T*, *R*, and *RH*, were identified up to 63 days pre-anthesis. However, no single predictor consistently maintained an association with $\hat{\lambda }$ during the entire study period. This framework advances the development of weather ‘markers’ for detailed environmental profiling of GEI drivers and improves upon prior approaches that limited window-pane analysis to disease outcomes from susceptible hosts to identify weather-based variables for predicting plant disease epidemics.

## Introduction

Weather exerts a substantial influence on the dynamics plant disease at different spatio-temporal scales. Temperature, humidity, and precipitation affect pathogen life-cycle processes, such as sporulation, dispersal and germination, while also modulating host defense mechanisms (Noel et al., 2024). This interplay among weather factors, often driven by mesoscale weather turbulences, is particularly critical during the lag phase of epidemics as it affects the onset, rate and intensity of disease (Schein, 1963). Weather also affects plant physiological processes that influence canopy development, flowering, and yield. Since crop plants are susceptible to disease, weather factors that affect pathogen reproduction and dispersal will influence whether disease will occur (Madden et al., 2007). Thus, weather factors that have been shown to be correlated with a disease outcome are subsequently used as predictors in models for predicting disease epidemics. However, designing and identifying useful weather-based predictor variables within the complex network of weather factors is challenging and has long been a key research topic in botanical epidemiology (Coakley et al., 1988; Shah et al., 2019). A common approach involves aggregating daily weather data over fixed calendar periods or key crop growth stages (Cucak et al., 2019; Mehra et al., 2017). However, fixed temporal frameworks may inadequately capture epidemic dynamics, since ecological processes are inherently fluid, and favorable conditions often arise intermittently. Thus, useful predictors of plant disease need to integrate both biological relevance (e.g., pathosystem-specific temperature thresholds) and critical temporal windows, because sporadic favorable conditions alone are rarely sufficient to trigger an epidemic.

Botanical epidemiologists commonly mine a time series of weather variables to identify periods and variables correlated with presence of disease (Carisse et al., 2018). A popular approach for aggregating weather data is the window-pane analysis, which identifies key time-window lengths or temporal ‘hotspots’ where weather variables are significantly associated with disease intensity during the growing season (Coakley et al., 1988; Kriss et al., 2010). This technique investigates statistical correlations between weather features, summarized within discrete fixed-length intervals, i.e., ‘window-pane’, and a disease outcome. By varying the window length, numerous overlapping windows are created. With the ending points of a window sliding along a time series, the aggregated variables themselves evolve into a time series. Further, applying feature engineering (Verdonck et al., 2024) to hourly weather data enables a high-resolution window-pane analysis that’s provides a fine-scale representation of environmental factors associated with a disease outcome (Dalla Lana et al., 2021a; Sanjel et al., 2024; Webster et al., 2023). However, one criticism of the window-pane is the multiple correlation tests that can lead to inflated Type I error rates due to the exhaustive search for associations in time (Shah et al., 2019). As the number of hypotheses tested increases, so does the risk of detecting false-positive correlations. To address this concern, several variable selection techniques such as the Simes’ method and machine learning variable mining have been adopted (Gouache et al., 2015; Kriss et al., 2010; Sanjel et al., 2024). In this study, we employ stability selection, a feature selection technique in machine learning that combines resampling methods with regularization (Bodinier et al., 2023; Meinshausen and Bühlmann, 2010), to identify sparse and non-redundant sets of weather-based predictors for plant disease.

Studies on weather-disease associations using window-pane analysis in botanical epidemiology typically use disease observations from selected susceptible cultivars in field surveys or variety performance trials as the response variable (Kriss et al., 2010). While this simplifies the analysis, it assumes that the identified weather patterns uniformly influence disease development across all cultivars. In addition, the selected susceptible cultivars may not represent cultivars planted by growers, and their performance may not reflect realistic field-level cultivar performance. Further, cultivar reaction to disease can vary in response to environmental factors, expressing genotype-by-environment interaction (GEI) that leads to differential responses of the same genotype across various environments (Garnica, 2024; Malosetti et al., 2013; Twizeyimana et al., 2008). Such location-specific cultivar rank changes are common in large multi-environment trials (MET) that lack universally susceptible checks and employ commercial cultivars that have undergone multiple selection cycles. Consequently, a highly susceptible cultivar in one environment may perform markedly differently in another environment. Thus, a more practical approach would be to model the GEI effect with a factor analytic (FA) linear mixed model (Resende et al., 2022; Smith et al., 2001) and extract appropriate model outputs for use as response variables in window-pane analysis. A FA model, similar to principal component analysis, partitions GEI effects into *k*th-order components: environmental loadings ( λ , representing environmental drivers), genotypic scores ( ς , representing plant genotype effects) and residual variances ( ψ ). Here, λ captures orthogonal environmental drivers that shape phenotypic responses, while still incorporating complete cultivar trial data. Values of λ can then be associated with weather variables to identify biologically relevant weather-based predictors for plant disease. This approach has been used to associate environmental variables to quantitative traits in crop (Azevedo et al., 2023; Rogers et al., 2021) and animal (Sae-Lim et al., 2014) systems.

In this study, we devise a high-resolution window-pane analytical framework to link hourly weather data to λ from a FA analysis of GEI effects in MET of plant disease response. This framework is applied to Stagonospora nodorum blotch (SNB) of winter wheat as a case study. The disease is caused by the fungus *Parastagonospora nodorum* and is prevalent in regions with warm, humid conditions and frequent rainfall (Shanner and Buechley, 1995). Yield losses of up to 30% have been reported in the Eastern and Pacific Northwest regions of the U.S., Western Australia, and Europe (Murray and Brennan, 2009). In the Southeastern U.S., severe SNB epidemics occur sporadically, with the disease being driven primarily by moderate temperatures and moist conditions. A risk model, developed based on accumulated temperature and moisture, performed well in predicting SNB onset in North Carolina (Mehra et al., 2017). However, the model was found to over-predict disease onset in the Piedmont region of the state (Adhikari et al., 2023a), highlighting a need to further refine weather predictors of SNB. Thus, the objective of this study was to identify weather-based predictors of SNB associated with λ using window-pane analysis augmented by stability selection. This framework is expected to improve on modeling efforts on SNB for accurate disease prediction and facilitate more targeted disease management strategies. Beyond SNB, this framework advances environmental profiling, offering potential for broader applications across agricultural systems where GEI plays a critical role in shaping biological outcomes of interest.

## Materials and methods

### Methodological overview

**Figure 1:**
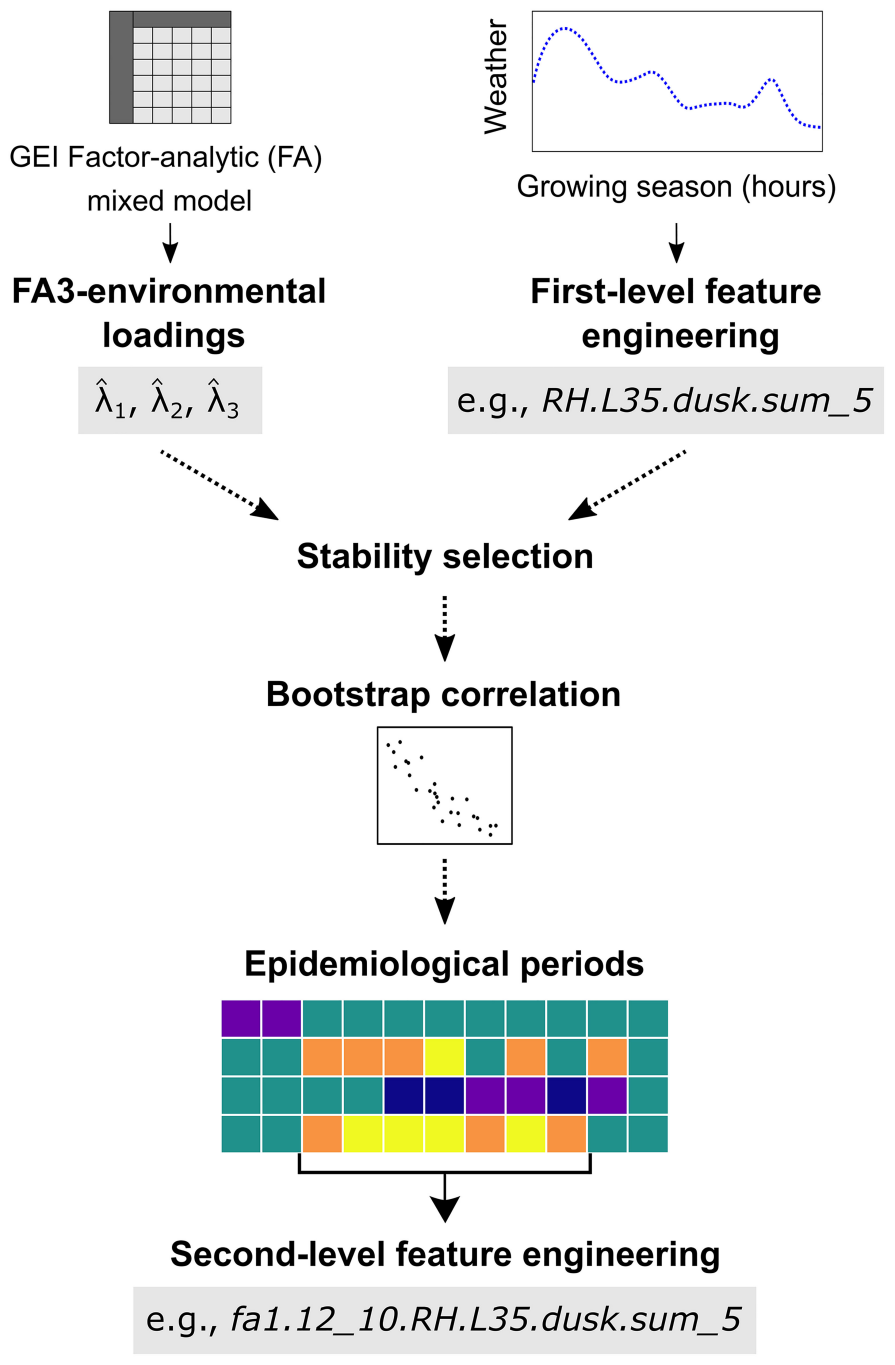
A schematic representation of the workflow adopted to identify weather-based variables associated with Stagonospora nodorum blotch in winter wheat from a multi-environmental trial. Briefly, environmental loading factors, $\hat{\lambda }$ , which are outputs of a third-order factor analytic (FA3) linear mixed model, are used as response variables. A first-level feature engineering is then used to create a matrix of time series variables. A stability selection algorithm is subsequently used to identify first-level weather-based variables consistently associated with $\hat{\lambda }\;$ in time and bootstrap correlation is then performed. Second-level feature engineering is used to aggregate significant first-level weather-based predictors over optimal epidemiological period and heatmaps used to visualize periods of continuous risk for each predictor.

A schematic representation of the workflow outlining the steps used to identify weather-based variables associated with plant disease is provided in **Figure 1**. The key steps can be summarized as follows: i) *Extraction of environmental loading*: obtaining rotated estimates of environmental loadings ( ${\hat{\lambda }}_{1},{\hat{\lambda }}_{2}$ , and ${\hat{\lambda }}_{3}$ ; collectively referred to as $\hat{\lambda }$ ) from the analysis of GEI using a factor analytic linear mixed model (FA). In this study, $\hat{\lambda }$ were obtained from FA analysis of SNB data collected from MET in North Carolina (Garnica, 2024); ii) *First-level feature engineering*: creating a matrix of first-level weather-based variables from hourly weather data based on the epidemiology of SNB; iii) *Stability selection*: applying the stability selection algorithm on the weather matrix to identify consistent associations between $\hat{\lambda }$ and first-level weather variables over time; iv) *Bootstrap correlation analysis*: perform bootstrap Spearman correlation analysis to evaluate the strength of stable associations between first-level weather-based variables and $\hat{\lambda }$ ; v) *Epidemiological periods*: visualizing periods of continuous disease risk via heatmaps, and vi) *Second-level feature engineering*: aggregating first-level weather predictors over optimal epidemiological period for each first-level weather variable to refine the library of predictors.

### Response variables

The dataset analyzed in this study, originally curated by Garnica (2024), is referred to as the ‘SNB dataset’. It comprises outputs of a third-order FA mixed model evaluating the performance and stability of 18 commercial winter wheat cultivars to SNB from 2021 to 2024 across 18 environments in North Carolina. These outputs are ${\hat{\lambda }}_{1},{\hat{\lambda }}_{2}$ , and ${\hat{\lambda }}_{3}$ and represent the estimated environmental loadings of the final SNB severity. Disease severity was expressed as the diseased leaf area (%), collected at Zadoks 75 – 80 growth stages (Zadoks et al., 1974). Essentially, these loadings represent latent environmental patterns that are linked to disease severity variation across different environments. Specifically, ${\hat{\lambda }}_{1}$ captures the dominant environmental gradient most strongly aligned with changes in disease severity, while ${\hat{\lambda }}_{2}$ and ${\hat{\lambda }}_{3}$ represent secondary and tertiary orthogonal environmental patterns, respectively. Thus, each environment in the ‘SNB dataset’ has three $\hat{\lambda }$ outputs as responses, resulting in a total of 54 (= 18 environments × 3 $\hat{\lambda }$ s) measurements that were used as responses in the study.

### First-level feature engineering

Hourly weather observations were obtained from on-site weather monitoring stations (WatchDog 1000 Series Micro Station, Spectrum Technologies, Aurora, IL) equipped with internal sensors for air temperature (*T*; °C; accuracy ± 0.6 °C), dew point (*D*; °C; accuracy ± 0.6 °C), precipitation (*R*; mm; resolution 0.2 mm), and relative humidity (*RH*; %; accuracy ± 3%). Sensors were positioned about 1.5-m above the soil surface in the center of each field site. Collected hourly weather data (***h*** =[*D*, *R*, *RH*, *T*]) extended from early-February (around 60 days before disease onset) to the last disease assessment date (around mid-May). This timeframe coincided with critical phases of SNB onset and development. Prior to data processing, the ***h*** time series was visually inspected to detect outliers and missing data. Data gaps due to sensor failure were filled-in with hourly weather records from the ECMWF, ERA5, and ERA5-Land reanalysis datasets accessed through the Open-Meteo API (Zippenfenig, 2023). Sensors malfunctioned at three of the eighteen sites for about 20 days during the growing season and the missing data were added based on the reanalysis records.

Drawing on previous studies exploring weather-disease associations (Dalla Lana et al., 2021a) and the sensitivity of weather sensors, ***h*** was engineered into various weather-based variables encompassing the mean, minima, maxima, cumulative summaries of hours meeting specific conditions, combinations of these summaries, and indices, such as dew point depression (*DPD*; °C), vapor pressure deficit (*VPD*, kPa), and growing degree days (*GDD*; °C) (**Table 1**). To explore the biological relevance of oscillations in *RH* and *T* on disease dynamics, a z-score peak detection algorithm was used. This algorithm triggers a signal (-1 or +1) when a new data point deviates by a set number of standard deviations from the moving average. The parameters are the *lag* (size of the moving average), *threshold* (z-score emitting signals), and *influence* (algorithm sensitivity; set to 0 in this study) (Brakel, 2014).

**Table 1:** Description of weather variables used to evaluate their association with environmental loading factors of a factor analytic model explaining genotype-by-environment interaction.

Abbreviation	Description
Relative humidity, (*RH*) %	
*RH.A*	Mean *RH*
*RH.max*	Maximum *RH*
*RH.min*	Minimum *RH*
*RH.AMP*	Daily amplitude in *RH*
*RH.L35*	Number of hours with *RH* ≤ 35%
*RH.G90*	Number of hours with *RH* ≥ 90%
*RH.40.rl.count6*	Number of events with at least 6 consecutive hours of *RH* ≤ 40%
*RH.90.rl.count6*	Number of events with at least 6 consecutive hours of *RH* ≥ 90%
*RH.40.rl.count8*	Number of events with at least 8 consecutive hours of *RH* ≤ 40%
*RH.90.rl.count8*	Number of events with at least 8 consecutive hours of *RH* ≥ 90%
*RH6.peak4*	Number of oscillations (positive and negative) in *RH* within a range of 4 standard deviation
Temperature (*T*), °C	
*T.A*	Mean *T*
*T.max*	Maximum *T*
*T.min*	Minimum *T*
*T.AMP*	Daily amplitude in *T*
*T.L0*	Number of hours with *T* ≤ 0°C
*T.3T7*	Number of hours with 3°C ≤ *T* ≤ 7°C
*T.7T10*	Number of hours with 7°C ≤ *T* ≤ 10°C
*T.10T13*	Number of hours with 10°C ≤ *T* ≤ 13°C
*T.13T16*	Number of hours with 13°C ≤ *T* ≤ 16°C
*T.16T19*	Number of hours with 16°C ≤ *T* ≤ 19°C
*T.19T22*	Number of hours with 19°C ≤ *T* ≤ 22°C
*T.22T25*	Number of hours with 22°C ≤ *T* ≤ 25°C
*T.25T28*	Number of hours with 25°C ≤ *T* ≤ 28°C
*T.G28*	Number of hours with *T* ≥ 28°C
*T6.peak4*	Number of oscillations (positive and negative) in *T* within a range of 4 standard deviations from a 6-h moving average
Precipitation (*R*), mm	
*R.S*	Total precipitation (mm)
*R.AH*	Number of hours with *R >* 0 mm
*R.1.rl.max*	Maximum number of consecutive hours with *R* ≥ 1 mm
*R.2.rl.max*	Maximum number of consecutive hours with *R* ≥ 2 mm
*R.0.5.rl.count5*	Number of events with at least 5 consecutive hours of *R* ≥ 0.5 mm
Combinations of *T* and *RH* (*TRH*)	
*TRH.16T19nRH.L40*	Number of hours with 16°C ≤ *T* ≤ 19°C and *RH* ≤ 40%
*TRH.19T22nRH.L40*	Number of hours with 19°C ≤ *T* ≤ 22°C and *RH* ≤ 40%
*TRH.22T25nRH.L40*	Number of hours with 22°C ≤ *T* ≤ 25°C and *RH* ≤ 40%
*TRH.25T28nRH.L40*	Number of hours with 25°C ≤ *T* ≤ 28°C and *RH* ≤ 40%
*TRH.G28nRH.L40*	Number of hours with *T* ≥ 28°C and *RH* ≤ 40%
*TRH.3T7nRH.G80*	Number of hours with 3°C ≤ *T* ≤ 7°C and *RH* ≥ 80%
*TRH.7T10nRH.G80*	Number of hours with 7°C ≤ *T* ≤ 10°C and *RH* ≥ 80%
*TRH.10T13nRH.G80*	Number of hours with 10°C ≤ *T* ≤ 13°C and *RH* ≥ 80%
*TRH.13T16nRH.G80*	Number of hours with 13°C ≤ *T* ≤ 16°C and *RH* ≥ 80%
*TRH.16T19nRH.G80*	Number of hours with 16°C ≤ *T* ≤ 19°C and *RH* ≥ 80%
*TRH.19T22nRH.G80*	Number of hours with 19°C ≤ *T* ≤ 22°C and *RH* ≥ 80%
Combinations of *T* and *R* (*TR*)	
*TR.3T7nR.G0.2*	Number of hours with 3°C ≤ *T* ≤ 7°C and *R* ≥ 0.2 mm
*TR.7T10nR.G0.2*	Number of hours with 7°C ≤ *T* ≤ 10°C and *R* ≥ 0.2 mm
*TR.10T13nR.G0.2*	Number of hours with 10°C ≤ *T* ≤ 13°C and *R* ≥ 0.2 mm
*TR.13T16nR.G0.2*	Number of hours with 13°C ≤ *T* ≤ 16°C and *R* ≥ 0.2 mm
*TR.16T19nR.G0.2*	Number of hours with 16°C ≤ *T* ≤ 19°C and *R* ≥ 0.2 mm
*TR.19T22nR.G0.2*	Number of hours with 19°C ≤ *T* ≤ 22°C and *R* ≥ 0.2 mm
Weather indices^a^	
*DPD*	Dew point depression (°C)
*VPD*	Vapor pressure deficit (kPa)
*GDD*	Sum of growing degree days (°C)

a *DPD* was calculated as described by Bosen (1958) as: $DPD=T-\left[\left(112+0.9T\right)RH^{0.125}-112+0.1T\right]$ , where *T* and *RH* are mean air temperature (°C) and relative humidity (%), respectively. *VPD* was calculated as: VPD=VPS−VPA , where $VPS=0.611\times 10^{\left(7.5D\right)/\left(237.3+D\right)}$ and VPA=VPS× RH/100 , where *D* is the dewpoint temperature (°C). *GDD* was calculated as $GDD=\left[\left(T_{max}+T_{min}\right)/2\right]–T_{base}$ , where *T_max_* and *T_min_* are maximum and minimum temperatures (°C) and *T_base_* was 0 °C (Kimball et al., 2012).

Weather-based variables were further summarized across various intra-day periods; 24-hour, daytime, nighttime, dawn (8-hour period starting 4 hours before sunrise), and dusk (8-hour period starting 3 hours before sunset). Intra-day periods were defined using site-specific sunrise and sunset times to account for seasonal variation (e.g., shorter nights in summer). This aimed to identify transitory variables associated with SNB dynamics. Weather variables were then aggregated into six rectangular (Pierre et al., 2021) rolling windows ( w ) of 5, 10, 15, 20, 25, and 30 days in length (i.e., $w_{5},\;w_{10},\;w_{15},\;w_{20},\;w_{25}$ , and $w_{30}$ , respectively). A total of 1,530 weather time series were generated from a combinations of ***h***, intra-day periods, and w . The naming convention of first-level weather variables is based on the weather element, feature engineering criteria, intra-day period, aggregation function (sum or mean), and the length of w . For example, *RH.L35.dusk.sum_5* represents the cumulative dusk hours with *RH* ≤ 35% over a 5-day rolling window.

### Reference point

Window-pane analysis was conducted as described by Dalla Lana et al. (2021a). The data processing and engineering processes above yielded an inventory of six datasets, one for each w . Each dataset included a vector of $\hat{\lambda }$ as the response variable and the matrix of first-level time series weather variables as independent variables. For correlation analysis, all series were synchronized to the predicted anthesis date (Zadoks 50 growth stage; i.e., *LAG* = 0), providing a standardized developmental timescale across location-years. Anthesis marks floral initiation in wheat and is often used as a reference point for various cultural practices, including timing of the last fungicide application in wheat (Paul et al., 2018).

The date of wheat anthesis in each environment was predicted using a modified version of the growth stage model by Zhao et al. (2021). This process-based model is a composite of four parameters (Equations 1–6) namely: i) nonlinear daily thermal time ( $\Delta TT_{d}$ , °C), ii) vernalization ( $fv_{d}$ , days), iii) photoperiod ( $fp_{d}$ , hours), and iv) a unitless temperature stress factor ( $Ts_{d})$ . The mathematical formulations underlying the prediction model for phenological events are described as follows:


(1) $$PVT_{d}=\sum_{d=1}^{t}\left(\Delta TT_{d}\;\times fv_{d}\;\times fp_{d}\times Ts_{d}\right)$$

where


(2) $$\Delta TT_{d}=\;\{\begin{array}{cc} 0 & T_{d}\leq 1.5\text{°C}\;or\;T_{d}\geq 37\text{°C}\\ 26*\left[exp\left(-{\left(\frac{T_{d}-26}{2\sigma_{p}}\right)}^{2}\right)\right] & 1.5\text{°C}<T_{d}\leq 26\text{°C}\\ 26*\left[1-{\left(\frac{T_{d}-26}{37-26}\right)}^{2}\right] & 26\text{°C}<T_{d}\leq 37\text{°C} \end{array}$$

(3) $$fv_{d}=\;\{\begin{array}{cc} 0 & VDD_{d}<30\;days\\ \frac{VDD_{d}-30}{80-30} & 30\;days<\;VDD_{d}\leq 80\;days\\ 1 & VDD_{d}>80\;days \end{array}$$

(4) $$VDD_{d}=\;\{\begin{array}{cc} 0 & T_{d}<-4\text{°C}\;or\;T_{d}>17\text{°C}\\ \frac{T_{d}-\left(-4\right)}{3-\left(-4\right)} & -4\text{°C}\leq \;T_{d}<3\text{°C}\\ 1 & 3\text{°C}\leq \;T_{d}<10\text{°C}\\ \frac{17-T_{d}}{10-17} & 10\text{°C}\leq T_{d}\leq 17\text{°C} \end{array}$$

(5) $$fp_{d}=\;\{\begin{array}{cc} 0 & ph_{d}<5\;hours\\ \frac{ph_{d}-5}{20-5} & 5\;hours<\;ph_{d}\leq 20\;hours\\ 1 & ph_{d}>20\;hours \end{array}$$

and


(6) $$Ts_{d}\;=\text{ sin}\left(\frac{\pi }{2}\frac{T_{d}-1.5}{26-1.5}\right)$$

In this model, $PVT_{d}$ (°C days) represents the accumulated thermal time from emergence (day 1) to day *t*, $T_{d}$ is the daily average *T*, $\sigma_{p}$ is a plant growth rate fixed at 7.6. The variable $VDD_{d}$ refers to accumulated vernalization days and $ph_{d}$ is the daily photoperiod. In this study, $\Delta TT_{d}$ began accumulating on October 10, 20, and 30 in the Piedmont, Southeastern Plains, and Middle Atlantic Coastal Plain region, respectively, reflecting regional differences in optimal wheat sowing timing in North Carolina (Post and Heiniger, 2021). An additional 148 degree days were added to $PVT_{d}$ to account for sowing-to-emergence phase (Zhao et al., 2021). Anthesis was reached when the adjusted accumulated $PVT_{d}$ at each environment exceeded 500 °C days. All weather data for the wheat anthesis model were sourced via the Open-Meteo API (Zippenfenig, 2023).

### Stability selection

Stability-based LASSO-regression was used to assess joint associations of SNB- λ metrics with time series weather variables. Stability selection is a feature selection technique in machine learning that combines resampling methods (e.g., bootstrapping) with regularized models to identify sparse, non-redundant sets of predictor variables (Meinshausen and Bühlmann, 2010). The method applies the LASSO regularization (Tibshirani, 1996) to each resampling iteration. For example, consider the dataset $D_{LAG}=\left(x_{ij},\;y_{j}\right)$ for one of the six w investigated, where each observation of $D_{LAG}$ is indexed by *LAG* (i.e., days relative to anthesis). In this dataset, $y_{j}$ is the vector of ${\hat{\lambda }}_{1_{j}}$ observed across *j* = 1, 2, …, *n* environments and $x_{ij}$ denotes the matrix of weather variables, indexed by *j* and *i* = 1, 2, …, *p*, where *p* represents the total number of first-level weather variables. The LASSO procedure applied on $D_{LAG}$ is defined as: $\text{argmin}\left\{\sum_{j=1}^{n}{\left(y_{j}-\sum_{i=1}^{p}{\text{β}}_{i_{\phi }}^{T}x_{ij}\right)}^{2}+\phi \sum_{i=1}^{p}\left|{\text{β}}_{i_{\phi }}\right|\right\}$ , where ϕ≥0 is a penalty parameter controlling the amount of shrinkage. LASSO executes variable selection by gradually shrinking the model parameters ${\text{β}}_{i}$ to zero as ϕ increases. In this study, we generated *B* = 1,000 bootstrap resampling datasets from $D_{LAG}$ . The selection probability, $\pi_{\phi }\left(i\right)$ , is calculated as: $\pi_{\phi }\left(i\right)=C_{\phi }\left(i\right)/B$ , where $C_{\phi }\left(i\right)$ is the number of times feature i is selected at penalty ϕ across over B bootstrap samples. The stability selection model $\left(V_{\phi ,\pi }\right)$ consists of features with $\pi_{\phi }\left(i\right)$ above a certain threshold π∈(0, 1) , defined as $V_{\phi ,\pi }=\left(i:\;\pi_{\phi }\left(i\right)\geq \pi \right)$ . Consequently, two tuning parameters (ϕ, π) for $V_{\phi ,\pi }$ need to be calibrated. The tuning parameters were calibrated by maximizing an internal stability score derived from the likelihood of uninformative feature selection (Bodinier et al., 2023). The resulting vector of stable first-level weather variable names was then used to subset the variables within $D_{LAG}$ for subsequent correlation analysis.

### Daily bootstrap correlation analysis

The degree of association between stable first-level weather-based variables and $\hat{\lambda }$ was examined using Spearman’s correlation test. This rank-based correlation method is resilient to outliers and is often used for non-linear associations of continuous variables. For each stable variable and *LAG* of $D_{LAG}$ , mean ( ${\hat{\rho }}^{*}$ ) and 95% confidence interval of estimates $\left[\hat{\rho }{*}_{lower},\hat{\rho }{*}_{upper}\right]$ were calculated from 1,000 bootstrap correlation samples. From the Central Limit Theorem, the sampling distribution of $\hat{\rho }$ approximates normality for sufficiently large sample sizes (Efron, 1982). Daily correlation analyses were conducted only when *j* ≥ 10.

### Second-level feature engineering

Window-pane analysis identifies relevant variables associated with response outcomes on a daily basis throughout the growing season. However, intermittent weather effects may not be sufficient to trigger biological processes that lead to an epidemic. To address this, we aggregated first-level variables that exhibited continuously significant daily associations (≥ 7 *LAGs* and $0\notin [\hat{\rho }{*}_{lower},\hat{\rho }{*}_{upper}]$ ) with $\hat{\lambda }$ , defining this as the optimal epidemiological period in the SNB etiology. For instance, if *RH.L35.dusk.sum_5* had an optimal epidemiological period for ${\hat{\lambda }}_{1}$ between *LAGs* 12 to 10, then the corresponding second-level variable would be named *fa1.12_10.RH.L35.dusk.sum_5* (**Figure 1**). In this naming convention, the prefix ‘*fa1*’ designates the first latent environmental factor ( ${\hat{\lambda }}_{1}$ ), while ‘*fa2*’ and ‘*fa3*’ correspond to the second ( ${\hat{\lambda }}_{2}$ ) and third ( ${\hat{\lambda }}_{3}$ ) environmental loadings, respectively. This systematic labeling facilitated tracking of feature derivation and biological relevance across the analytical framework. Further, if in a given environment, *RH.L35.dusk.sum_5* recorded 9, 18, 14, 5, 3, 2, and 1 hour(s) from *LAG*s 15 and 9, the aggregated second-level variable, *fa1.12_10.RH.L35.dusk.sum_5*, was calculated as 10 hours (= 5 + 3 + 2), representing the variable values at *LAG*s 12, 11, and 10. This methodology was systematically applied across all environments to generate distributions for each second-level numerical weather-based predictor. A description of weather-based variables examined in this study is presented in **Table 1**.

### Performance of second-level weather-based predictors

To evaluate the performance of identified second-level weather-based variables as potential predictors of SNB severity, the average SNB severity was calculated for all environments in the ‘SNB dataset’ (Garnica, 2024). For selected second-level weather-based variables, the average SNB severity was then plotted against cumulative hours (or events) of each weather-based variable over the optimum epidemiological period. Pearson correlation analysis was then used to determine the direction and strength of the linear relationship between the average SNB severity and cumulative hours (or events) for each variable.

### Software and code availability

Reproducible scripts and documentation related to this study are available at https://github.com/vcgarnica/SNB_window_pane. The code was written in R Studio (version 2024.04.2) and executed in R (version 4.4.1) (R Core Team, 2024). Stability selection and bootstrap correlation analyses were conducted on the North Carolina State University Hazel High-Performance Computing Cluster. Custom shell and R scripts managed job execution, specifying core count, memory, local directory, and the R script. Correlation results for each w and $\hat{\lambda }$ were saved as RData objects and imported back into R Studio for visualization. Key R packages used included *furrr* (Vaughan and Dancho, 2022), *future* (Bengtsson, 2021), *lubridate* (Grolemund and Wickham, 2011), *meteor* for obtaining site-specific photoperiod (Hijmans et al., 2023), *openmeteo* for filling weather gaps and predicting anthesis date (Pisel, 2023), *rstatix* for correlation analysis (Kassambara, 2023), *sharp* for stability selection (Bodinier, 2023), *suncalc* for site-specific sunset and sunrise hours (Thieurmel and Elmarhraoui, 2022), and *tidyverse* (Wickham et al., 2019).

## Results

### Prediction of anthesis date

Anthesis date, defined as the day of year (DOY) when most wheat cultivars in each environment began flowering, served as the reference point for the window-pane analysis. The observed anthesis date ranged from DOY 100 in KS23 to DOY 118 in ROX24 (**Table 2**), with an average date of DOY 108 across all environments. Averages of observed anthesis date by region were DOY 110 (Piedmont), DOY 104 (Southeastern Plains), and DOY 108 (Middle Atlantic Coastal Plain). The model predicted anthesis within ±5 days of the observed dates for about 83% of the environments, except in LB23, SB23, and SB24, where deviations of -8, +7 and +9 days, respectively, occurred (**Table 2**). Averages of the predicted anthesis date within a region were within ±1 day of the observed date of anthesis across all the regions.

**Table 2:** Observed and predicted date of wheat anthesis in each environment in the ‘SNB dataset’ used to associate weather-based variables to Stagonopora nodorum blotch in multi-environment trials.

		Date of anthesis			
Region^a^	Environment^a^	Observed (mm/dd/yy)	Observed (DOY)^b^	Predicted (DOY)^b^	ΔDOY^c^
Piedmont	AL24	4/15/2024	106	109	3
Piedmont	CL22	4/24/2022	114	114	0
Piedmont	MR22	4/22/2022	112	111	-1
Piedmont	OX23	4/22/2023	112	111	-1
Piedmont	RO24	4/27/2024	118	116	-2
Piedmont	SB22	4/23/2022	113	115	2
Piedmont	SB23	4/14/2023	104	111	7
Piedmont	SB24	4/15/2024	106	115	9
Piedmont	UN23	4/20/2023	110	105	-5
Southeastern Plains	KS22	4/17/2022	107	107	0
Southeastern Plains	KS23	4/10/2023	100	104	4
Southeastern Plains	KS24	4/13/2024	104	107	3
Southeastern Plains	LB23	4/18/2023	108	100	-8
Southeastern Plains	LB24	4/18/2024	109	105	-4
Southeastern Plains	RW22	4/13/2022	103	103	0
Middle Atlantic Coastal Plains	BE24	4/13/2024	104	109	5
Middle Atlantic Coastal Plains	PY22	4/20/2022	110	112	2
Middle Atlantic Coastal Plains	PY23	4/22/2023	112	107	-5

a A description of geographic regions and environments is provided in Garnica, (2024).

b DOY denotes the day of the year.

c Difference between the observed date of anthesis and predicted date; predicted data was determined based on a modified version of a mechanistic model by Zhao et al. (2021).

### Descriptive analysis of weather-based variables

A total of 1,307 first-level weather-based variables exhibited stable associations with $\hat{\lambda }$ for at least one day within the study period, with ${\hat{\rho }}^{*}$ values ranging from -0.93 to 0.92. Many variables were associated with multiple $\hat{\lambda }$ components. For example, 1,038 variables were associated with ${\hat{\lambda }}_{1}$ , 1,111 variables with ${\hat{\lambda }}_{2}$ , and 929 variables with ${\hat{\lambda }}_{3}$ . While association strengths were consistent across components, temporal patterns differed markedly, with most variables exhibiting sporadic, single-day correlations rather than sustained relationships over multiple days. For visualization, we considered only weather-based variables showing continuous associations (i.e., ≥ 7 *LAGs*) associated with ${\hat{\lambda }}_{1}$ , ${\hat{\lambda }}_{2}$ , and ${\hat{\lambda }}_{3}$ (see details below). A complete set of first-level weather variables associated with each $\;\hat{\lambda }\;$ can be accessed in the project’s associated GitHub repository https://github.com/vcgarnica/SNB_window_pane. Below, we describe results for each $\hat{\lambda }$ with particular emphasis on ${\hat{\lambda }}_{1}$ , which is the dominant loading factor explaining most of the environmental variations in SNB epidemics (Garnica, 2024).

**Figure 2:**
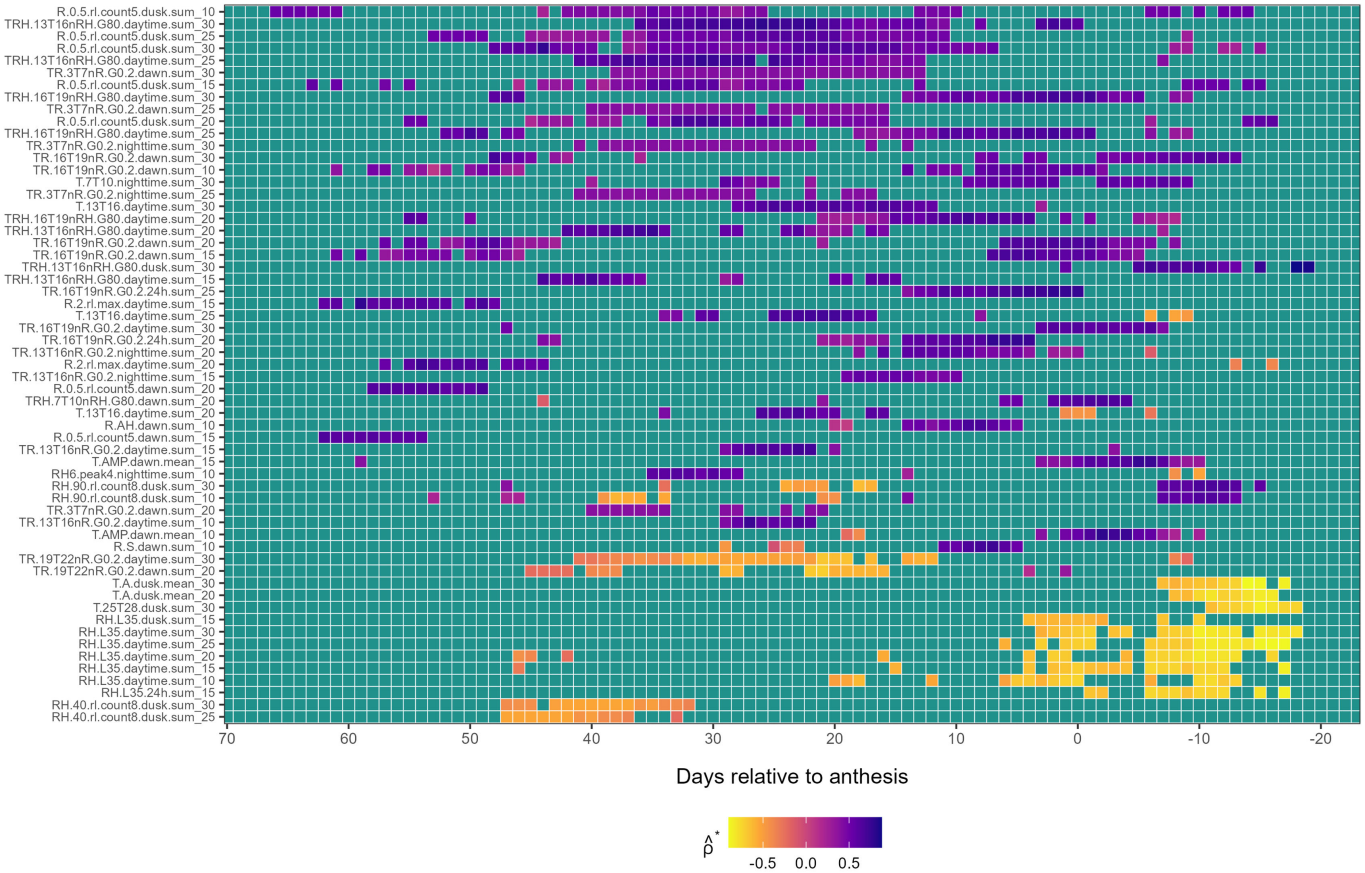
Heatmap illustrating the association of first-level weather variable (*y-axis*) with the first environmental loading factor, ${\hat{\lambda }}_{1}$ , during the growing season (*x-axis*), relative to the anthesis date (*LAG* = 0). The response variable, ${\hat{\lambda }}_{1}$ , is from a factor analysis of foliar severity of Stagonospora nodorum blotch in winter wheat in a multi-environment trial. The *y-axis* is limited to weather-based variables displaying continuously significant associations (≥ 7 *LAGs* and $0\notin \left[\hat{\rho }{*}_{lower},\hat{\rho }{*}_{upper}\right]$ ) with ${\hat{\lambda }}_{1}.\;$ Colors in the heatmap denote the strength of Spearman correlation ( ${\hat{\rho }}^{*}$ ), ranging from -1 (yellow) to +1 (blue).

*First-level weather-based variables associated with* ${\hat{\lambda }}_{1}$ : Persistent associations of first-level weather-based variables with ${\hat{\lambda }}_{1}$ were predominantly positive, except in a few cases involving the families *TR.19T22nR.G0.2* and *RH.40.rl.count8* at pre-anthesis and *RH.L35* and *T.A* at post-anthesis (**Figure 2**). The earliest and most prolonged associations of variables with ${\hat{\lambda }}_{1}$ were observed about 45 days pre-anthesis and involved three variable families; *TRH.13T16nRH.G80*, *R.0.5.rl.count5*, and *TR.3T7nR.G0.* For example, the variable *R.0.5.rl.count5.dusk.sum_10* exhibited a moderately positive association with ${\hat{\lambda }}_{1}$ from *LAG* 47 to 24. Variables of this same family, such as *R.0.5.rl.count5.dusk.sum_25* and *R.0.5.rl.count5.dusk.sum_30*, displayed different and more continuous temporal patterns (**Figure 2**). The variable *TRH.13T16nRH.G80.daytime.sum_30* exhibited a stronger and less intermittent positive association with ${\hat{\lambda }}_{1}$ from *LAG* 37 to 9. Further, *TR.3T7nR.G0.2.dawn.sum_30* exhibited a persistent and positive association from *LAG* 37 to 12. Additional positive associations were observed near anthesis with the *TR.16T19nR.G0.2* and *TRH.16T19nRH.G80* families. Further, weather variables reflecting oscillations in *RH* (e.g., *RH6.peak4*), were positively associated 30 days pre-anthesis. Additional variables, including those derived from *R.S* and *T.AMP* conditions at dawn, also displayed positive associations with ${\hat{\lambda }}_{1}$ around anthesis (**Figure 2**).

A few negative associations between first-level weather-based variables and ${\hat{\lambda }}_{1}$ were also detected, especially post-anthesis (**Figure 2**). The earliest negative associations were observed between *LAG* 56 and 32 involving variables from the family *RH.40.rl.count8.dusk* at different w . Mid-season, negative associations were identified for the family *TR.19T22nR.G0.2* at different w between *LAG* 42 to 10 days pre-anthesis. The strongest negative associations were found post-anthesis with family *RH.L35*. For example, *RH.L35.daytime.sum_30* exhibited a negative association with ${\hat{\lambda }}_{1}$ post-anthesis from *LAG* 5 to -18 (**Figure 2**). Indices based on *DPD*, *GDD*, and *VPD* did not exhibit continuous significant associations with ${\hat{\lambda }}_{1}$ .

**Figure 3:**
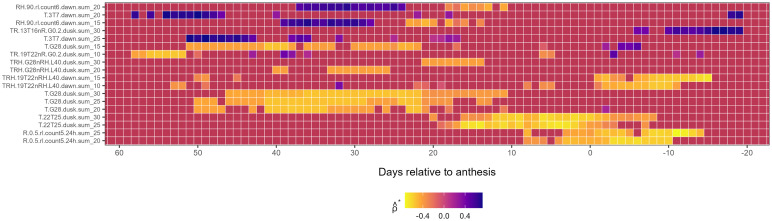
Heatmap illustrating the association of first-level weather variable (*y-axis*) associated with the second environmental loading factor, ${\hat{\lambda }}_{2}$ , during the growing season (*x-axis*), relative to the anthesis date (*LAG* = 0). The response variable, ${\hat{\lambda }}_{2}$ , is from a factor analysis of foliar severity of Stagonospora nodorum blotch in winter wheat in a multi-environment trial. The *y-axis* is limited to weather-based variables displaying continuously significant associations (≥ 7 *LAGs* and $0\notin \left[\hat{\rho }{*}_{lower},\hat{\rho }{*}_{upper}\right]$ ) with ${\hat{\lambda }}_{2}.\;$ Colors in the heatmap denote the strength of Spearman correlation ( ${\hat{\rho }}^{*}$ ), ranging from -1 (yellow) to +1 (blue).

*First-level weather variables associated with* ${\hat{\lambda }}_{2}$ : Unlike for ${\hat{\lambda }}_{1}$ , associations between first-level weather-based variables and ${\hat{\lambda }}_{2}$ were predominantly negative (**Figure 3**), with only a few exceptions (e.g., *RH6.peak4.dusk*, *RH.90.rl.count6.dawn*, and *T.3T7.dawn*, each occurring at different temporal scales and w ). Early and strong negative associations with ${\hat{\lambda }}_{2}\;$ were observed for *TR.19T22nR.G0.2.dusk* (from *LAG* 58 to 50), *T.G28.dusk* (from *LAG* 50 to 10) and intermittently for *TRH.G28nRH.L40.dusk* (from *LAG* 42 to 21). Among these variables, the *T.G28.dusk* family exhibited the most prolonged negative association with ${\hat{\lambda }}_{2}$ , while *TR.19T22nR.G0.2.dusk* exhibited the strongest correlation with ${\hat{\lambda }}_{2}\;$ near anthesis and post-anthesis. Precipitation variables such as *R.0.5.rl.count5.24h* at $w_{25}$ and $w_{20}$ and temperature-precipitation combinations *TRH.19T22nRH.L40.dawn* at $w_{15}$ and $w_{10}$ also showed negative correlations for more than 10 consecutive days around and post-anthesis (**Figure 3**).

**Figure 4:**
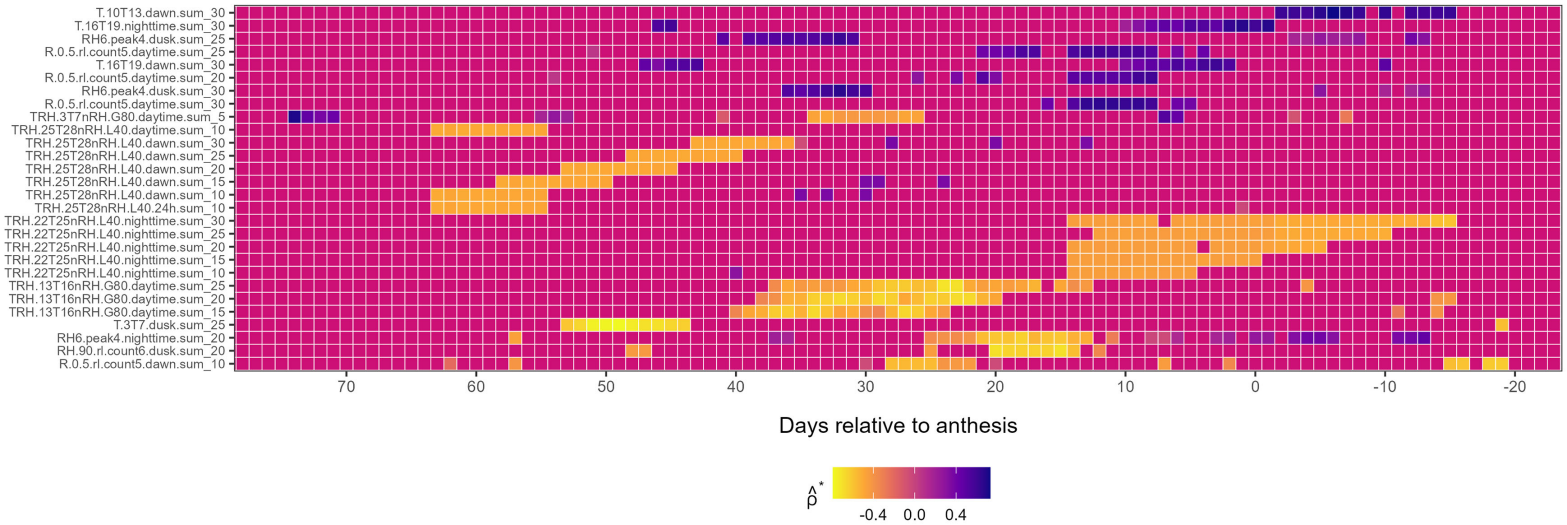
Heatmap illustrating the association of first-level weather variable (*y-axis*) associated with the third environmental loading factor, ${\hat{\lambda }}_{3}$ , during the growing season (*x-axis*), relative to the anthesis date (*LAG* = 0). The response variable, ${\hat{\lambda }}_{3}$ , is from a factor analysis of foliar severity of Stagonospora nodorum blotch in winter wheat in a multi-environment trial. The *y-axis* is limited to meteorological variables displaying continuously significant associations (≥ 7 *LAGs* and $0\notin \left[\hat{\rho }{*}_{lower},\hat{\rho }{*}_{upper}\right]$ ) with ${\hat{\lambda }}_{3}.\;$ Colors in the heatmap denote the strength of Spearman correlation ( ${\hat{\rho }}^{*}$ ), ranging from -1 (yellow) to +1 (blue).

*First-level weather variables associated with* ${\hat{\lambda }}_{3}$ : First-level weather variables associated with ${\hat{\lambda }}_{3}$ exhibited a mix of positive and negative correlations (**Figure 4**). Early-season negative associations with ${\hat{\lambda }}_{3}$ were observed for the *TRH.25T28nRH.L40.dawn* (from *LAG* 64 to 36) and *T.3T7.dusk* (from *LAG* 55 to 43) families. Mid-season, negative associations with ${\hat{\lambda }}_{3}$ were observed for *TRH.13T16nRH.G80.daytime* and *TRH.10T13nRH.G80.dawn* families from *LAG* 40 and 12, while post-anthesis, the *TRH.22T25nRH.L40.nighttime* family was associated with ${\hat{\lambda }}_{3}$ (**Figure 4**). Further, the variable *RH6.peak4.nighttime.sum_20* was negatively associated with ${\hat{\lambda }}_{3}$ from *LAG* 25 to 8 pre-anthesis, while *T.16T19.nighttime.sum_30* was positively associated with ${\hat{\lambda }}_{3}$ in the 7 days leading up to anthesis (**Figure 4**).

### Library of second-level weather-based variables

Window-pane analysis involved two stages: first, identifying daily associations between first-level variables and $\hat{\lambda }$ during the study period (earl-February to mid-May), as described above; second, consolidating first-level variables into optimal epidemiological periods relative to anthesis to design second-level weather-based variables. To qualify as a second-level variable, a variable had to exhibit a significant and continuous correlation (≥ 7 *LAGs* and $0\notin \left[\hat{\rho }{*}_{lower},\hat{\rho }{*}_{upper}\right]$ ) with $\hat{\lambda }\;$ during optimal epidemiological periods and a 3-day separation between periods. Based on these criteria, 60, 19, and 28 second-level weather-based variables were identified for ${\hat{\lambda }}_{1}$ (**Table 3**), ${\hat{\lambda }}_{2}$ (**Supplementary Table S1**), and ${\hat{\lambda }}_{3}$ (**Supplementary Table S2**). Variables from the *TRH*, *T*, and *RH* families exhibited a more Gaussian-like distribution, while those related to *R* and *TR* conditions were more skewed. Below, we highlight some second-level weather variables that were associated with ${\hat{\lambda }}_{1}$ , ${\hat{\lambda }}_{2}$ , and ${\hat{\lambda }}_{3}$ .

**Table 3:** Description of weather-based variables associated with the first environmental loading factor, ${\hat{\lambda }}_{1}$ .

First-level weather-based variable	*LAG*		Duration (days)	Second-level weather-based variable	Descriptive statistics^a^ (hours or events)					
	*Start*	*End*			NA^b^	min	q1	mean	q3	max
Pre-anthesis										
* R.0.5.rl.count5.dawn.sum_15*	62	54	9	*fa1.62_54.R.0.5.rl.count5.dawn.sum_15*	6	0	0	3.3	9	9
* R.2.rl.max.daytime.sum_15*	59	52	7	*fa1.59_52.R.2.rl.max.daytime.sum_15*	4	0	2	11.1	15.5	32
* R.0.5.rl.count5.dawn.sum_20*	58	49	11	*fa1.58_49.R.0.5.rl.count5.dawn.sum_20*	6	0	0	4.2	10	10
* R.2.rl.max.daytime.sum_20*	55	49	7	*fa1.55_49.R.2.rl.max.daytime.sum_20*	4	0	1.8	11.6	17.8	31
* RH.40.rl.count8.dusk.sum_25*	47	40	7	*fa1.47_40.RH.40.rl.count8.dusk.sum_25*	1	0	0	2.8	8	8
* TRH.13T16nRH.G80.daytime.sum_15*	44	37	7	*fa1.44_37.TRH.13T16nRH.G80.daytime.sum_15*	0	16	74.2	110.4	133.5	240
* RH.40.rl.count8.dusk.sum_30*	43	35	8	*fa1.43_35.RH.40.rl.count8.dusk.sum_30*	2	0	0	3.3	9	9
* R.0.5.rl.count5.dusk.sum_10*	42	30	11	*fa1.42_30.R.0.5.rl.count5.dusk.sum_10*	0	0	0	1.4	3	8
* TRH.13T16nRH.G80.daytime.sum_20*	42	34	8	*fa1.42_34.TRH.13T16nRH.G80.daytime.sum_20*	0	28	115.2	154.6	179	298
* TR.3T7nR.G0.2.nighttime.sum_25*	41	27	9	*fa1.41_27.TR.3T7nR.G0.2.nighttime.sum_25*	0	0	0	4.4	0	36
* TRH.13T16nRH.G80.daytime.sum_25*	41	18	25	*fa1.41_18.TRH.13T16nRH.G80.daytime.sum_25*	0	183	334.8	431.1	491.2	836
* TR.3T7nR.G0.2.dawn.sum_20*	40	34	11	*fa1.40_34.TR.3T7nR.G0.2.dawn.sum_20*	0	0	0	2.6	0	28
* TR.3T7nR.G0.2.dawn.sum_25*	40	16	25	*fa1.40_16.TR.3T7nR.G0.2.dawn.sum_25*	0	0	0	13.8	0	105
* TR.3T7nR.G0.2.nighttime.sum_30*	39	22	10	*fa1.39_22.TR.3T7nR.G0.2.nighttime.sum_30*	1	0	0	6.7	0	60
* R.0.5.rl.count5.dusk.sum_15*	38	30	15	*fa1.38_30.R.0.5.rl.count5.dusk.sum_15*	0	0	0	1.6	3	9
* TR.3T7nR.G0.2.dawn.sum_30*	38	13	26	*fa1.38_13.TR.3T7nR.G0.2.dawn.sum_30*	1	0	0	17.4	0	140
* TRH.13T16nRH.G80.daytime.sum_30*	36	13	25	*fa1.36_13.TRH.13T16nRH.G80.daytime.sum_30*	0	244	376.2	508.5	592.8	978
* RH6.peak4.nighttime.sum_10*	35	28	8	*fa1.35_28.RH6.peak4.nighttime.sum_10*	0	6	14.2	27.4	37.8	47
* TR.19T22nR.G0.2.daytime.sum_30*	32	19	14	*fa1.32_19.TR.19T22nR.G0.2.daytime.sum_30*	0	0	10.2	35.7	45.8	130
* TR.13T16nR.G0.2.daytime.sum_15*	29	22	8	*fa1.29_22.TR.13T16nR.G0.2.daytime.sum_15*	0	0	6.2	15.3	20.8	60
* R.0.5.rl.count5.dusk.sum_25*	28	11	17	*fa1.28_11.R.0.5.rl.count5.dusk.sum_25*	0	0	0	5.9	12.8	22
* T.13T16.daytime.sum_30*	28	12	18	*fa1.28_12.T.13T16.daytime.sum_30*	0	685	915.8	1,138.80	1,354.00	1,534.00
* TR.13T16nR.G0.2.daytime.sum_10*	28	22	8	*fa1.28_22.TR.13T16nR.G0.2.daytime.sum_10*	0	0	0	8.4	14.8	44
* T.13T16.daytime.sum_20*	26	20	7	*fa1.26_20.T.13T16.daytime.sum_20*	0	182	243.2	328.9	399	473
* R.0.5.rl.count5.dusk.sum_30*	25	7	7	*fa1.25_7.R.0.5.rl.count5.dusk.sum_30*	0	0	0	7.4	16.2	26
* T.13T16.daytime.sum_25*	25	17	14	*fa1.25_17.T.13T16.daytime.sum_25*	0	296	401.2	517.2	626.5	702
* R.0.5.rl.count5.dusk.sum_20*	22	16	8	*fa1.22_16.R.0.5.rl.count5.dusk.sum_20*	0	0	0	1.9	5.2	7
* TR.19T22nR.G0.2.dawn.sum_20*	22	16	8	*fa1.22_16.TR.19T22nR.G0.2.dawn.sum_20*	0	0	1.2	13.6	17.5	51
* TR.13T16nR.G0.2.nighttime.sum_15*	19	10	9	*fa1.19_10.TR.13T16nR.G0.2.nighttime.sum_15*	0	0	0	18.1	28.8	82
* TRH.16T19nRH.G80.daytime.sum_20*	15	4	9	*fa1.15_4.TRH.16T19nRH.G80.daytime.sum_20*	0	58	98.2	134.5	167.8	231
* TR.13T16nR.G0.2.nighttime.sum_20*	14	5	10	*fa1.14_5.TR.13T16nR.G0.2.nighttime.sum_20*	0	0	0	19.2	29.5	90
* TR.16T19nR.G0.2.24h.sum_20*	14	4	7	*fa1.14_4.TR.16T19nR.G0.2.24h.sum_20*	0	20	45	67.2	83.8	124
* TR.16T19nR.G0.2.24h.sum_25*	12	0	12	*fa1.12_0.TR.16T19nR.G0.2.24h.sum_25*	0	40	62	96.1	112.8	192
* R.AH.dawn.sum_10*	11	5	11	*fa1.11_5.R.AH.dawn.sum_10*	0	0	21.2	35	41.2	111
* R.S.dawn.sum_10*	11	5	8	*fa1.11_5.R.S.dawn.sum_10*	0	0	18	62.9	93.7	235.7
* T.7T10.nighttime.sum_30*	9	2	7	*fa1.9_2.T.7T10.nighttime.sum_30*	0	350	400.8	458.8	506	604
* RH.L35.daytime.sum_10*	6	0	7	*fa1.6_0.RH.L35.daytime.sum_10*	0	0	13.5	54.4	87.5	126
Post-anthesis										
* TRH.16T19nRH.G80.daytime.sum_30*	13	-5	19	*fa1.13_-5.TRH.16T19nRH.G80.daytime.sum_30*	0	165	244.2	318.3	382.5	515
* TRH.16T19nRH.G80.daytime.sum_25*	12	-1	14	*fa1.12_-1.TRH.16T19nRH.G80.daytime.sum_25*	0	96	141.2	191.6	246	319
* TR.16T19nR.G0.2.dawn.sum_10*	8	-2	11	*fa1.8_-2.TR.16T19nR.G0.2.dawn.sum_10*	0	0	0	9.8	13.2	52
* TR.16T19nR.G0.2.dawn.sum_15*	7	-2	10	*fa1.7_-2.TR.16T19nR.G0.2.dawn.sum_15*	0	0	0	13.1	20.5	60
* TR.16T19nR.G0.2.dawn.sum_20*	6	-2	9	*fa1.6_-2.TR.16T19nR.G0.2.dawn.sum_20*	0	0	0	15.2	25.8	60
* RH.L35.dusk.sum_15*	4	-2	7	*fa1.4_-2.RH.L35.dusk.sum_15*	0	0	9.5	32.8	52	78
* TR.16T19nR.G0.2.daytime.sum_30*	3	-6	10	*fa1.3_-6.TR.16T19nR.G0.2.daytime.sum_30*	0	4	20.2	35.9	49	74
* RH.L35.daytime.sum_15*	2	-12	15	*fa1.2_-12.RH.L35.daytime.sum_15*	0	0	41	134.1	188.5	351
* TRH.7T10nRH.G80.dawn.sum_20*	2	-4	7	*fa1.2_-4.TRH.7T10nRH.G80.dawn.sum_20*	0	35	71.5	103.3	130.8	222
* T.AMP.dawn.mean_10*	0	-6	7	*fa1.0_-6.T.AMP.dawn.mean_10*	0	18.1	53.2	55.4	62.4	68.5
* T.AMP.dawn.mean_15*	0	-7	8	*fa1.0_-7.T.AMP.dawn.mean_15*	0	19.7	58	61.1	68.7	75.8
* T.7T10.nighttime.sum_30*	-2	-9	8	*fa1.-2_-9.T.7T10.nighttime.sum_30*	2	286	313.2	383.8	451.8	506
* TR.16T19nR.G0.2.dawn.sum_30*	-4	-13	10	*fa1.-4_-13.TR.16T19nR.G0.2.dawn.sum_30*	1	0	0	27.5	38	117
* TRH.13T16nRH.G80.dusk.sum_30*	-5	-13	9	*fa1.-5_-13.TRH.13T16nRH.G80.dusk.sum_30*	1	19	72	107	142	180
* RH.L35.24h.sum_15*	-6	-12	7	*fa1.-6_-12.RH.L35.24h.sum_15*	2	0	9.5	61.9	92.5	167
* RH.L35.daytime.sum_20*	-6	-13	8	*fa1.-6_-13.RH.L35.daytime.sum_20*	2	0	15.8	95	150.5	215
* RH.L35.daytime.sum_25*	-6	-17	12	*fa1.-6_-17.RH.L35.daytime.sum_25*	2	0	29	167.9	257	490
* RH.90.rl.count8.dusk.sum_10*	-7	-13	7	*fa1.-7_-13.RH.90.rl.count8.dusk.sum_10*	2	0	0	1.6	1.8	7
* RH.90.rl.count8.dusk.sum_30*	-7	-13	7	*fa1.-7_-13.RH.90.rl.count8.dusk.sum_30*	2	0	0	5	7	21
* RH.L35.daytime.sum_30*	-7	-13	7	*fa1.-7_-13.RH.L35.daytime.sum_30*	2	12	43.5	136.2	193.2	462
* T.A.dusk.mean_30*	-7	-15	9	*fa1.-7_-15.T.A.dusk.mean_30*	2	70.1	155.9	154.8	166.2	172.1
* T.A.dusk.mean_20*	-8	-16	9	*fa1.-8_-16.T.A.dusk.mean_20*	2	53.4	154.3	153.6	171.3	176.6
* T.25T28.dusk.sum_30*	-11	-18	8	*fa1.-11_-18.T.25T28.dusk.sum_30*	3	24	72	122.6	171.5	203

a NA is the number of missing values, min = minimum, q1 = first quartile, q3 = third quartile, and max = maximum values. ‘*Count*’-based variables (e.g., *R.0.5.rl.count5.dusk.sum_15*) and ‘*peak*’-based variables (e.g., *RH6.peak4.nighttime.sum_10*) measure the number of events during the optimal period, while other variables indicate accumulated hours.

b Missing values in descriptive summary at the beginning and end of the season was due to variations in the length of the growing season among environments.

For each first-level variable, the start and end of the optimal epidemiological period (defined as days relative to predicted wheat anthesis, *LAG*), along with its duration and descriptive statistics for the corresponding second-level aggregated variables, are presented.

The variable *fa1.62_54.R.0.5.rl.count5.dawn.sum_15* was one of the second-level weather-based variables associated with ${\hat{\lambda }}_{1}$ early in the season. This variable exhibited associations with ${\hat{\lambda }}_{1}$ about 62 days pre-anthesis and with events ranging from 0 to 9 (mean = 3.3) across environments (**Table 3**). Prior to anthesis, variables that exhibited associations with ${\hat{\lambda }}_{1}$ over the longest epidemiological period included *fa1.41_18.TRH.13T16nRH.G80.daytime.sum_25* that showed associations for 24 continuous days, with values ranging from 183 to 836 hours across environments. This variation in accumulated hours represents about 3.3% to 14.4% of the possible 6,250 hours in this window (assuming a 10-hour daytime period over a 25-day window). The other variable was *fa1.38_13.TR.3T7nR.G0.2.dawn.sum_30* that exhibited associations with ${\hat{\lambda }}_{1}$ for 26 days with values ranging from 0 to 140 hours (mean = 17.4). The variable *fa1.28_12.T.13T16.daytime.sum_30* was associated with ${\hat{\lambda }}_{1}$ for 18 days and accumulated up to a maximum of 1,534 hours, the highest among all the variables examined (**Table 3**). Post-anthesis, the variables *fa1.-2_-9.T.7T10.nighttime.sum_30* and *fa1.-6_-13.RH.L35.daytime.sum_20* were also associated with ${\hat{\lambda }}_{1}$ , accumulating up to 506 and 215 hours, respectively, over an 8-day epidemiological period (**Table 3**).

Pre-anthesis, *fa2.46_22.T.G28.dusk.sum_25* was associated with ${\hat{\lambda }}_{2}$ over the longest epidemiological period of 25 days, accumulating a maximum of 39 hours (mean = 4.6) (**Supplementary Table S1**), reflecting a low but significant contribution of dusk hours with *T* ≥ 28°C to disease severity variation. In contrast, *fa2.51_44.T.3T7.dawn.sum_25* was associated with ${\hat{\lambda }}_{2}$ for only 8 days but accumulated a maximum of 443 hours in a single environment, one of the highest totals within this group of variables. Among variables associated with ${\hat{\lambda }}_{3}$ , which accounted for the smallest portion of environmental variation driving cultivar-specific SNB responses across environments, *fa3.31_22.TRH.13T16nRH.G80.daytime.sum_25* was associated with ${\hat{\lambda }}_{3}$ as early as 31 days before anthesis, accumulating as much as 413 hours in one environment. The variable *fa3.14_-15.TRH.22T25nRH.L40.nighttime.sum_30* exhibited the longest association with ${\hat{\lambda }}_{3}$ post-anthesis, spanning a 30-day optimal epidemiological period and reaching 29 hours in the highest environment (**Supplementary Table S2**).

### Performance of selected weather variables

**Figure 5:**
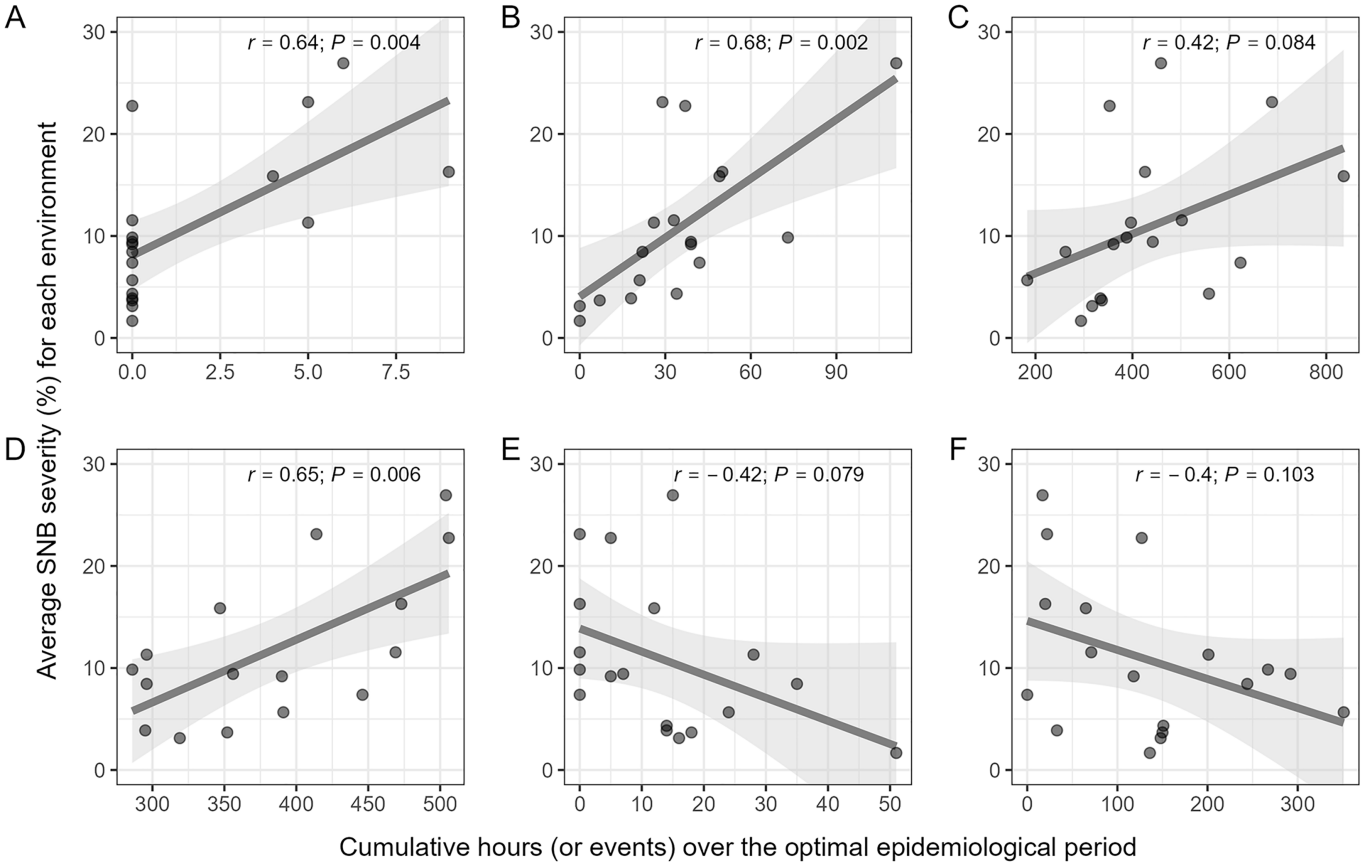
Scatterplot of values of six selected second-level weather-based variables associated with the first environmental loading factor ( ${\hat{\lambda }}_{1}$ ) and the average SNB severity (%) across environments in the SNB dataset. **(A)** *fa1.38_30.R.0.5.rl.count5.dusk.sum_15*, i.e., cumulative number of dusk events with at least 5 consecutive hours of precipitation ≥ 0.5 mm over a 15-day rolling window from *LAG* 38 to 30 (e.g., days relative to the anthesis date); **(B)** *fa1.11_5.R.AH.dawn.sum_10*, i.e., the cumulative number of precipitation events over a 10-day rolling window from *LAG* 11 to 5; **(C)** *fa1.-2_-9.T.7T10.nighttime.sum_30*, i.e., the cumulative number of nighttime hours with air temperature between 7 – 10 °C over a 30-day rolling window from *LAG* - 2 to -9; **(D)** *fa1.41_18.TRH.13T16nRH.G80.daytime.sum_25*, i.e., cumulative number of daytime hours with air temperature between 13 – 16 °C and relative humidity ≥ 80%, over a 25-day rolling window from *LAG* 41 to 18; **(E)** *fa1.22_16.TR.19T22nR.G0.2.dawn.sum_20*, i.e., the cumulative number of dawn hours with air temperature between 16 – 19 °C over a 20-day rolling window from *LAG* 22 to 16; and **(F)** *fa1.2_-12.RH.L35.daytime.sum_15*, i.e., the cumulative number of daytime hours with relative humidity ≤ 35%, over a 15-day rolling window from *LAG* 2 to -12. Pearson correlation coefficient (*r*) and its associated *P*-value measure the strength of association between levels of a weather-based variables and average SNB severity. The smoothed line represents predicted values and its corresponding confidence interval (gray area) generated by fitting a linear model with the *geom_smooth* function in the R package *ggplot2*.

Cumulative hours (or events) of selected second-level weather-based variables associated with ${\hat{\lambda }}_{1}$ were correlated with SNB severity but the direction and strength of that association varied among the variables (**Figure 5**). Among variables that were positively associated with disease severity, significant correlations were observed for *fa1.38_30.R.0.5.rl.count5.dusk.sum_15* (*r* = 0.64; *P* = 0.004) (**Figure 5A**), *fa1.11_5.R.AH.dawn.sum_10* (*r* = 0.68; *P* = 0.002) (**Figure 5B**), and *fa1.41_18.TRH.13T16nRH.G80.daytime.sum_25 (r* = 0.65*; P* = 0.006*)* (**Figure 5D**), while the correlation for *fa1.-2_-9.T.7T10.nighttime.sum_30* was marginally non-significant (*r* = 0.46; *P* = 0.082) (**Figure 5C**). In contrast, correlations for *fa1.22_16.TR.19T22nR.G0.2.dawn.sum_20* (*r* = −0.42; *P* = 0.079) (**Figure 5E**) were negative and marginally non-significant, and that of *fa1.2_-12.RH.L35.daytime.sum_15* (*r* = −0.40; *P* = 0.103) (**Figure 5F**) was negative and non-significant.

## Discussion

Plant disease prediction models are integral components of decision support systems that help growers evaluate epidemic risk and the need for intervention to prevent disease from economically impacting yield (Madden et al., 2007; González-Domínguez et al., 2023). These models are driven by weather variables reflecting conditions that favor disease development at critical crop developmental stages during the season. In most cases, weather variables from research linking weather conditions to a disease outcome are used as predictors in these models. However, designing and identifying suitable weather-based predictors continues to be a significant challenge (Shah et al., 2019). This challenge could be due to the stochastic nature of weather, the complex interactions among weather variables and how this affects both the host and the pathogen, confounding effects of host resistance on disease expression under varying weather conditions, and the resolution of weather data used to design predictors. To address these limitations, this study developed an environmental profiling pipeline to identify weather-based predictors associated with GEI effects in MET. This framework captures potential weather-driven GEI factors contributing to variability in disease severity across environments and genotypes. A high-resolution window-pane analysis was augmented with stability selection to identify robust first level predictors associated with $\hat{\lambda }$ , that were aggregated over optimal epidemiological periods to generate second-level variables. Using SNB of wheat as a case study, several second-level weather-based predictors that were significantly associated with ${\hat{\lambda }}_{1}$ were identified. Further, second-level weather variables such as *fa1.38_30.R.0.5.rl.count5.dusk.sum_15*, *fa1.11_5.R.AH.dawn.sum_10* and *fa1.41_18.TRH.13T16nRH.G80.daytime.sum_25* were strongly correlated with disease severity. The identified weather-based variables could be useful predictors in models that assess the risk of SNB. To the best of our knowledge, this is the first study that provides a framework to design and identify weather-based predictors of plant disease associated with latent environmental effects from analysis of MET using high-throughput weather data.

Studies in botanical epidemiology that use window-pane analysis to identify weather-based predictors of disease, have typically relied on correlations between time series of weather variables with disease from a susceptible cultivar (Coakley et al., 1988; Dalla Lana et al., 2021a; Kriss et al., 2010; Pietravalle et al., 2003; Sanjel et al., 2024). However, the use of disease from a susceptible cultivar as a response variable does not accurately represent the expected host response to the pathogen, since it is a product of genetic, environmental and experimental noise. Further, disease outcome is limited to a single environment within which a trial is conducted. In this present study, $\hat{\lambda }$ , an estimated parameter output from a FA model fitted to disease observations collected from a cultivar performance MET, was used as the response variable. As such, $\hat{\lambda }$ provides a more generalized measure of the environmental qualities on disease development, devoid of genetic factors, while incorporating data from all test cultivars. In this study, $\hat{\lambda }$ was composed of three estimates of rotated environmental loadings, i.e., ${\hat{\lambda }}_{1},{\hat{\lambda }}_{2}$ , and ${\hat{\lambda }}_{3}$ . The first loading ${\hat{\lambda }}_{1}$ is the dominant factor and second-level weather-based variables associated with ${\hat{\lambda }}_{1}$ are expected to be the primary determinants of weather-driven variations in the severity of SNB. In contrast, ${\hat{\lambda }}_{2}$ and ${\hat{\lambda }}_{3}$ played secondary roles, with varying levels depending on the level of association with specific weather variables. The use of $\hat{\lambda }$ as a response variable in studies on weather-disease associations offers a better characterization of the effect of environment on disease, potentially improving inference of the influence of weather on a disease outcome. Thus, this approach provides an advantage over previous approaches that solely utilize raw disease data from susceptible genotypes to identify weather predictors of disease using window-pane analysis.

The relationship between weather and disease is complex due to the potentially high number of non-linear associations and variable interactions among weather factors during the growing season (Cunniffe et al., 2015). Incorporating higher-order $\hat{\lambda }$ components ( ${\hat{\lambda }}_{2}$ and ${\hat{\lambda }}_{3}$ ) into the analysis, enabled uncovering of cultivar-specific responses to temperature regimes. For example, variables such as *fa2.51_44.T.3T7.dawn.sum_25* (i.e., accumulated hours with dawn temperatures between 3 - 7°C in late winter/early spring) and *fa2.12_-1.T.22T25.dusk.sum_30* (i.e., accumulated hours with dusk temperatures between 22 - 25°C near anthesis) were associated with ${\hat{\lambda }}_{2}$ , contributing to cultivar rank changes in SNB susceptibility across environments. Temperature-sensitive SNB resistance has been reported previously (Kim and Bockus, 2003; Da Luz and Bergstrom, 1986). For instance, the reaction of wheat cultivar AGSECO 7853 to SNB shifted from susceptible at cooler temperatures (10 - 18°C) to moderately resistant at warmer temperatures (21 - 29°C), while Heyne maintained resistance across all temperatures, and Newton remained susceptible (Kim and Bockus, 2003). This differential response may reflect temperature-modulated expression of susceptibility genes like *Snn1*, a wall-associated kinase with demonstrated temperature sensitivity (Noel et al., 2024; Shi et al., 2016). Analogous mechanisms occur in the wheat-*Puccinia striiformis* pathosystem (Feng et al., 2018). Our empirical analyses suggest that temperature regimes linked to ${\hat{\lambda }}_{2}$ and ${\hat{\lambda }}_{3}$ appear to affect host physiology or pathogen virulence, driving environment-dependent variation in cultivar performance in MET. Thus, incorporating the corresponding variables into predictive models could improve GEI resolution and accuracy of SNB prediction models at the landscape level.

In this study, many of the identified weather-based predictors associated with $\hat{\lambda }$ were not simple 24-hour summaries, but rather intra-day conditions such as dawn, dusk, and even nighttime, depending on the order of $\hat{\lambda }$ . This may be because daily averages mask fine-scale weather variations that influence *P. nodorum* processes such as spore production, deposition, and infection. El Jarroudi et al. (2017) and Bernard et al. (2022) reported that intra-day variations and oscillations in *T* and *RH* were positively associated with the development of Septoria leaf blotch of wheat caused by *Zymoseptoria tritici* but did not establish the time when these oscillations coincided with disease development. Results of the present study indicate these oscillations may play a role in the development of SNB epidemics pre-anthesis. More specifically, the variable *fa1.35_28.RH6.peak4.nighttime.sum_10*, which was associated with ${\hat{\lambda }}_{1}$ for 8 continuous days ~30 days pre-anthesis and *fa3.39_31.RH6.peak4.dusk.sum_25*, which was associated with ${\hat{\lambda }}_{3}$ for 9 continuous days ~40 days pre-anthesis and other ${\hat{\lambda }}_{3}$ -related variables, were found to influence disease dynamics. These findings are consistent with observations by Scharen (1966), who reported that drying and wetting cycles were conducive to the production of pycnidia and the release of pycnidiospores of *Septoria nodorum*. Thus, we expected to see a greater contribution of oscillations in *RH* and *T* earlier in the season. We indeed observed a strong positive association between another *RH* oscillation variable (*RH8.peak4.nighttime*) and ${\hat{\lambda }}_{1}$ for around 3 weeks about 50 days pre-anthesis, based on the 2022 and 2023 data. However, this association weakened when the 2024 data was incorporated into the analysis. Additional studies are needed to better understand the role of these *RH* oscillations in the risk of SNB, particularly during tillering and stem elongation.

Weather conducive to SNB development occurred in discrete patterns rather than as continuous trends during the growing season. For example, the frequency of precipitation events lasting 5 hours or more with ≥ 0.5 mm of rainfall (e.g., *fa1.62_54.R.0.5.rl.count5.dawn.sum_15*), as early as 60 days pre-anthesis, was associated with ${\hat{\lambda }}_{1}$ . In contrast, some weather-based variables representing combinations of *T* and *RH* were associated with ${\hat{\lambda }}_{1}$ both pre-anthesis and post-anthesis. For example, first-level variables such as *TRH.13T16nRH.G80.daytime.sum* and *TRH.16T19nRH.G80.daytime.sum* were positively associated with ${\hat{\lambda }}_{1}$ early in the season pre-anthesis and post-anthesis, depending on the rolling-day window used to summarize the weather data. Thus, careful considerations are needed when selecting appropriate weather-based variables as disease predictors. Predicting risk of disease pre-anthesis is important in wheat, and thus weather-based variables significantly associated with ${\hat{\lambda }}_{1}$ early in the season will probably be more appropriate. The significant association between ${\hat{\lambda }}_{1}\;$ and weather-based variables representing combinations of *T* and *RH*, is supported by the observation that for SNB, *RH* interacts with temperature, with *RH* having a stronger effect on lesion expansion under higher temperatures than at lower temperatures (Adhikari et al., 2023b). Weather-based variables such as *R.0.5.rl.count5.dusk.sum_30* and *TRH.13T16nRH.G80.daytume.sum_25*, which persisted for over longer periods during the season, were not always strongly associated with $\;{\hat{\lambda }}_{1}$ during the corresponding time periods. In contrast, variables such *R.0.5.rl.count5.dusk.sum_20* and *TRH.13T16nRH.G80.daytime.sum_25* that persisted for a relatively shorter time were strongly associated with ${\hat{\lambda }}_{1}$ within the corresponding temporal window. In most cases, intermittent weather effects may not be sufficient to trigger biological processes that lead to an epidemic. Thus, optimal epidemiological periods were defined to identify first-level variables that exhibited continuous significant association with ${\hat{\lambda }}_{1}$ . This step ensured that the resultant second-level weather-based variables would have effects that are adequate to trigger disease.

As latent environmental metrics, $\hat{\lambda }$ represent unknown components that collectively capture influences of the environment, including weather, biological, soil and other uncharacterized factors, on disease. Since field trials in this study were conducted in the same locations across different years, variations in $\hat{\lambda }$ within the SNB dataset were thus predominantly driven by weather. This observation seems reasonable since genetic variation in *P. nodorum* populations across the region is low (Kaur et al., 2024). Weather variables were differentially associated with $\hat{\lambda }$ , with each loading factor absorbing complementary weather signals. The dominant loading factor ${\hat{\lambda }}_{1}$ was associated with weather-based variables that generally enhanced disease risk, indicating an overall environmental predisposition to increase risk of disease. In contrast, ${\hat{\lambda }}_{2}$ and ${\hat{\lambda }}_{3}$ were associated with variables that reduced disease risk and likely captured environment-drive variation in cultivar response. To improve robustness against temporal offsets in weather-disease relationships, we aggregated stable first-level variables (those with single-day associations with $\hat{\lambda }\;$ components) over key epidemiological periods to create second-level predictors. This approach provides an advantage over plant disease prediction methods that rely solely on first-level weather variables (Dalla Lana et al., 2021b), as it enhances the likelihood of detecting weather drivers even when the optimal epidemiological window shifts by 1 – 2 days. However, the presence of autocorrelation in these second-level features may inflate the importance of the predictors as described below. This arises because the moving window approach aggregates information across overlapping time intervals, meaning that consecutive windows often include many of the same days. As a result, the derived variables are not temporally independents, since the values for adjacent windows are correlated due to shared underlying weather patterns. This redundancy can bias effect size estimates or variable selection processes by over-representing temporally clustered environmental signals. Disentangling this autocorrelation is at best challenging, due to the operations involving adjacent days within the optimal epidemiological period. The matrix of weather-based predictors identified in this study is analogous to DNA markers of plant genotypes, and can be used to describe the ‘E’ component of the GEI, casting multiple weather markers supporting wide-scale environmental prediction (Costa-Neto et al., 2021; Resende et al., 2022). This matrix could also be valuable for predicting disease risk as it optimizes decisions across environments that may never have been experimentally tested (Li et al., 2021; Piepho and Williams, 2024).

A granular feature engineering approach was used to identify intricate weather-based variables associated with SNB risk. This process produced a matrix of highly correlated elements, with over 1,500 time series evaluated daily for correlation with $\hat{\lambda }\;$ during the growing season. Conducting such an extensive number of hypothesis tests may lead to spurious correlations and increased Type I error rate. Previous studies have handled this complexity in various ways. Kriss et al. (2010) employed the Simes’ method (Simes, 1986), while Gouache et al. (2015) applied a three-step variable selection method combining elastic-net, cross-validation, and classic stepwise selection to reduce the number of weather variables examined. Others adopted a combination of biologically meaningful criteria and expert knowledge to select weather factors (Pietravalle et al., 2003; te Beest et al., 2009). Sanjel et al. (2024) applied LASSO regression to the window-pane data and used cross validation to tune the penalty parameter controlling the degree of shrinkage. In this study, we employed stability selection (Meinshausen and Bühlmann, 2010; Shah and Samworth, 2012), which combines LASSO regularization and resampling techniques, and an internal stability score (Bodinier et al., 2023) to automate variable selection. Unlike traditional dimensionality reduction methods, stability selection focuses on reproducibly detecting interactions across data subsets, reducing the number of testable hypotheses. To minimize the number of hypothesis tests, effective feature engineering through careful selection of summary metrics (e.g., means, minima, maxima or their combinations) is applied to align with biologically meaningful thresholds and the sensitivity of the weather sensors. Further, while there have been some valid criticisms with the window-pane analysis, specifically with the use of fixed-length windows for variable aggregation (Shah et al., 2019), our approach generated interpretable predictors that captured distinct temporal dimensions of SNB dynamics. For instance, variable families differed in unique predictors, with some families yielding a few predictors (e.g., *RH6.peak4.nighttime*, *R.S.dawn*, *RH.40.rl.count8.dusk*), while others (e.g., *RH.L35.daytime*, *RH.L35.daytime*) recurring across time windows. This balance of diversity and redundancy demonstrates the capacity of the method to capture both broad and granular weather signals.

In summary, there is growing interest in mining field-level weather data for plant disease predictive modeling (Dalla Lana et al., 2021b; Shah et al., 2019). This study examined a framework to detect and quantify associations between weather variables and metrics describing environmental components of GEI effects in MET, using SNB as a case study. More specifically, since GEI effects on SNB foliar severity were predominantly non-crossover (i.e., strictly positive ${\hat{\lambda }}_{1}$ ) (Garnica, 2024), weather variables derived from $\;{\hat{\lambda }}_{1}$ are expected to influence disease severity uniformly across all cultivars, irrespective of the cultivar susceptibility profile. In contrast, variables derived from ${\hat{\lambda }}_{2}$ and ${\hat{\lambda }}_{3}$ , will likely influence cultivar-specific rank changes across environments. The latter reflects the differential response of cultivars to the local environment. Incorporating these weather variables in prediction models will likely result in accurate estimates of the actual risk of disease outbreak, which will help growers better determine the need for intervention. The methodology described in this study can also be customized to generate weather predictors for other host-pathogen systems with similar attributes as SNB, provided there is sufficient knowledge on weather factors driving the dynamics of the disease of interest.

## Data Availability

Our original data availability statement was written as follows: R scripts and datasets are hosted at https://github.com/vcgarnica/SNB_window_pane.
