# Supplementary material for: Leveraging window-pane analysis with environmental factor loadings of genotype-by-environment interaction to identify high-resolution weather-based variables associated with plant disease
Source: Front Plant Sci. 2025 Sep 11;16:1637130. doi: 10.3389/fpls.2025.1637130 (PMC12460299; doi:10.3389/fpls.2025.1637130)
Supplement: Supplementary file 1 [file Table1.docx]

**TABLE S1**

Description of the 19 weather-based variables associated with the second environmental loading factor, $\hat{\lambda}_{2}$, in the ‘SNB dataset’. For each first-level variable, the optimal epidemiological period, defined by the start and end days relative to predicted wheat anthesis (*LAG*), along with its duration and descriptive statistics for the corresponding second-level aggregated variables, are presented.

| First-level variable | *LAG* | | Duration  (days) | Second-level variable | Descriptive statistics^a^ (hours or events) | | | | | | |
| --- | --- | --- | --- | --- | --- | --- | --- | --- | --- | --- | --- |
|  | *Start* | *End* |  |  | NA^b^ | min | q1 | mean | q3 | max | |
| Pre-anthesis | | | | | | | | | | |  |
| *TR.19T22nR.G0.2.dusk.sum_10* | 58 | 52 | 7 | *fa2.58_52.TR.19T22nR.G0.2.dusk.sum_10* | 1 | 0 | 0.0 | 0.7 | 0.0 | 7 | |
| *T.3T7.dawn.sum_20* | 54 | 48 | 7 | *fa2.54_48.T.3T7.dawn.sum_20* | 4 | 75 | 191.0 | 223.6 | 261.0 | 326 | |
| *T.3T7.dawn.sum_25* | 51 | 44 | 8 | *fa2.51_44.T.3T7.dawn.sum_25* | 4 | 109 | 242.5 | 293.2 | 344.8 | 443 | |
| *T.G28.dusk.sum_15* | 51 | 38 | 14 | *fa2.51_38.T.G28.dusk.sum_15* | 0 | 0 | 0.0 | 2.1 | 0.0 | 26 | |
| *T.G28.dusk.sum_25* | 46 | 22 | 25 | *fa2.46_22.T.G28.dusk.sum_25* | 1 | 0 | 0.0 | 4.6 | 0.0 | 39 | |
| *T.G28.dusk.sum_30* | 46 | 22 | 25 | *fa2.46_22.T.G28.dusk.sum_30* | 4 | 0 | 0.0 | 3.5 | 0.0 | 49 | |
| *T.G28.dusk.sum_20* | 41 | 28 | 14 | *fa2.41_28.T.G28.dusk.sum_20* | 0 | 0 | 0.0 | 4.3 | 0.0 | 35 | |
| *RH.90.rl.count6.dawn.sum_15* | 38 | 29 | 10 | *fa2.38_29.RH.90.rl.count6.dawn.sum_15* | 0 | 6 | 14.2 | 32.9 | 41.8 | 71 | |
| *RH.90.rl.count6.dawn.sum_20* | 36 | 24 | 13 | *fa2.36_24.RH.90.rl.count6.dawn.sum_20* | 0 | 14 | 31.0 | 51.4 | 69.0 | 97 | |
| *TRH.G28nRH.L40.dusk.sum_20* | 33 | 26 | 8 | *fa2.33_26.TRH.G28nRH.L40.dusk.sum_20* | 0 | 0 | 0.0 | 1.3 | 0.0 | 16 | |
| *T.G28.dusk.sum_15* | 30 | 22 | 9 | *fa2.30_22.T.G28.dusk.sum_15* | 0 | 0 | 0.0 | 1.7 | 0.0 | 19 | |
| *TRH.G28nRH.L40.dusk.sum_30* | 21 | 14 | 8 | *fa2.21_14.TRH.G28nRH.L40.dusk.sum_30* | 0 | 0 | 0.0 | 1.3 | 0.0 | 16 | |
| *T.22T25.dusk.sum_25* | 16 | 9 | 8 | *fa2.16_9.T.22T25.dusk.sum_25* | 0 | 70 | 109.8 | 124.9 | 145.5 | 165 | |
| Post-anthesis | | | | | | | | | | |  |
| *T.22T25.dusk.sum_30* | 12 | -1 | 14 | *fa2.12_-1.T.22T25.dusk.sum_30* | 0 | 218 | 277.2 | 322.8 | 367.8 | 453 | |
| *R.0.5.rl.count5.24h.sum_20* | 3 | -10 | 14 | *fa2.3_-10.R.0.5.rl.count5.24h.sum_20* | 0 | 0 | 10.0 | 15.4 | 17.8 | 56 | |
| *R.0.5.rl.count5.24h.sum_25* | 3 | -13 | 17 | *fa2.3_-13.R.0.5.rl.count5.24h.sum_25* | 0 | 0 | 15.0 | 19.8 | 26.8 | 67 | |
| *TRH.19T22nRH.L40.dawn.sum_10* | -2 | -11 | 10 | *fa2.-2_-11.TRH.19T22nRH.L40.dawn.sum_10* | 0 | 0 | 0.0 | 1.4 | 0.8 | 10 | |
| *TRH.19T22nRH.L40.dawn.sum_15* | -6 | -15 | 10 | *fa2.-6_-15.TRH.19T22nRH.L40.dawn.sum_15* | 2 | 0 | 0.0 | 1.9 | 0.8 | 10 | |
| *TR.13T16nR.G0.2.dusk.sum_30* | -11 | -19 | 9 | *fa2.-11_-19.TR.13T16nR.G0.2.dusk.sum_30* | 3 | 0 | 0.0 | 4.2 | 9.0 | 12 | |

^a^ NA is the number of missing values, min = minimum, q1 = first quartile, q3 = third quartile, and max = maximum values. ‘*Count*’-based variables (e.g., *R.0.5.rl.count5.24h.sum_25*) and ‘*peak*’-based variables (e.g., *RH6.peak4.dusk.sum_25*) measure the number of events during the optimal period, while other variables measure accumulated hours.

^b^ Missing values in descriptive summary at the beginning and end of the season was due to variations in the length of the growing season among environments.

**TABLE S2**

Description of the 28 weather-based variables associated with the third environmental loading factor, $\hat{\lambda}_{3}$, in the ‘SNB dataset’. For each first-level variable, the optimal epidemiological period, defined by the start and end days relative to predicted wheat anthesis (*LAG*), along with its duration and descriptive statistics for the corresponding second-level aggregated variables, are presented.

| First-level variable | *LAG* | | Duration  (days) | Second-level variable | Descriptive statistics^a^ (hours or events) | | | | | |
| --- | --- | --- | --- | --- | --- | --- | --- | --- | --- | --- |
|  | *Start* | *End* |  |  | NA^b^ | min | q1 | mean | q3 | max |
| Pre-anthesis | | | | | | | | | |  |
| *TRH.25T28nRH.L40.24h.sum_10* | 63 | 55 | 9 | *fa3.63_55.TRH.25T28nRH.L40.24h.sum_10* | 2 | 0 | 0.0 | 0.6 | 0.0 | 9 |
| *TRH.25T28nRH.L40.dawn.sum_10* | 63 | 55 | 9 | *fa3.63_55.TRH.25T28nRH.L40.dawn.sum_10* | 2 | 0 | 0.0 | 0.6 | 0.0 | 9 |
| *TRH.25T28nRH.L40.daytime.sum_10* | 63 | 55 | 9 | *fa3.63_55.TRH.25T28nRH.L40.daytime.sum_10* | 2 | 0 | 0.0 | 0.6 | 0.0 | 9 |
| *TRH.25T28nRH.L40.dawn.sum_15* | 58 | 50 | 9 | *fa3.58_50.TRH.25T28nRH.L40.dawn.sum_15* | 2 | 0 | 0.0 | 0.6 | 0.0 | 9 |
| *T.3T7.dusk.sum_25* | 53 | 44 | 10 | *fa3.53_44.T.3T7.dusk.sum_25* | 6 | 49 | 85.0 | 149.9 | 182.8 | 363 |
| *TRH.25T28nRH.L40.dawn.sum_20* | 53 | 45 | 9 | *fa3.53_45.TRH.25T28nRH.L40.dawn.sum_20* | 2 | 0 | 0.0 | 0.6 | 0.0 | 9 |
| *TRH.25T28nRH.L40.dawn.sum_25* | 48 | 40 | 9 | *fa3.48_40.TRH.25T28nRH.L40.dawn.sum_25* | 2 | 0 | 0.0 | 0.6 | 0.0 | 9 |
| *TRH.25T28nRH.L40.dawn.sum_30* | 43 | 36 | 8 | *fa3.43_36.TRH.25T28nRH.L40.dawn.sum_30* | 2 | 0 | 0.0 | 0.5 | 0.0 | 8 |
| *RH6.peak4.dusk.sum_25* | 39 | 31 | 9 | *fa3.39_31.RH6.peak4.dusk.sum_25* | 0 | 31 | 73.2 | 88.1 | 106.0 | 127 |
| *TRH.13T16nRH.G80.daytime.sum_15* | 39 | 31 | 9 | *fa3.39_31.TRH.13T16nRH.G80.daytime.sum_15* | 0 | 48 | 89.2 | 122.3 | 141.2 | 229 |
| *RH6.peak4.dusk.sum_30* | 36 | 30 | 7 | *fa3.36_30.RH6.peak4.dusk.sum_30* | 0 | 34 | 70.8 | 82.7 | 100.2 | 122 |
| *TRH.13T16nRH.G80.daytime.sum_20* | 36 | 21 | 16 | *fa3.36_21.TRH.13T16nRH.G80.daytime.sum_20* | 0 | 144 | 178.8 | 239.9 | 259.8 | 460 |
| *TRH.3T7nRH.G80.daytime.sum_5* | 34 | 26 | 9 | *fa3.34_26.TRH.3T7nRH.G80.daytime.sum_5* | 0 | 0 | 0.0 | 1.7 | 0.0 | 25 |
| *TRH.13T16nRH.G80.daytime.sum_25* | 31 | 22 | 10 | *fa3.31_22.TRH.13T16nRH.G80.daytime.sum_25* | 0 | 107 | 133.0 | 188.7 | 208.8 | 413 |
| *R.0.5.rl.count5.dawn.sum_10* | 28 | 22 | 7 | *fa3.28_22.R.0.5.rl.count5.dawn.sum_10* | 0 | 0 | 0.0 | 0.6 | 0.0 | 7 |
| *RH6.peak4.nighttime.sum_20* | 21 | 13 | 9 | *fa3.21_13.RH6.peak4.nighttime.sum_20* | 0 | 21 | 38.8 | 48.2 | 59.2 | 81 |
| *RH.90.rl.count6.dusk.sum_20* | 20 | 14 | 7 | *fa3.20_14.RH.90.rl.count6.dusk.sum_20* | 0 | 0 | 1.2 | 7.9 | 13.0 | 17 |
| *R.0.5.rl.count5.daytime.sum_20* | 14 | 8 | 7 | *fa3.14_8.R.0.5.rl.count5.daytime.sum_20* | 0 | 0 | 0.0 | 4.8 | 7.0 | 14 |
| *R.0.5.rl.count5.daytime.sum_25* | 14 | 8 | 7 | *fa3.14_8.R.0.5.rl.count5.daytime.sum_25* | 0 | 0 | 0.0 | 5.5 | 7.8 | 17 |
| *R.0.5.rl.count5.daytime.sum_30* | 14 | 8 | 7 | *fa3.14_8.R.0.5.rl.count5.daytime.sum_30* | 0 | 0 | 0.0 | 6.3 | 11.5 | 21 |
| *TRH.22T25nRH.L40.nighttime.sum_10* | 14 | 5 | 10 | *fa3.14_5.TRH.22T25nRH.L40.nighttime.sum_10* | 0 | 0 | 0.0 | 1.1 | 0.0 | 10 |
| *TRH.22T25nRH.L40.nighttime.sum_15* | 14 | 0 | 15 | *fa3.14_0.TRH.22T25nRH.L40.nighttime.sum_15* | 0 | 0 | 0.0 | 1.7 | 0.0 | 15 |
| *T.16T19.dawn.sum_30* | 8 | 2 | 7 | *fa3.8_2.T.16T19.dawn.sum_30* | 0 | 216 | 295.2 | 325.0 | 355.8 | 418 |
| Post-anthesis | | | | | | | | | |  |
| *TRH.22T25nRH.L40.nighttime.sum_20* | 14 | -5 | 20 | *fa3.14_-5.TRH.22T25nRH.L40.nighttime.sum_20* | 2 | 0 | 0.0 | 2.4 | 0.0 | 19 |
| *TRH.22T25nRH.L40.nighttime.sum_30* | 14 | -15 | 30 | *fa3.14_-15.TRH.22T25nRH.L40.nighttime.sum_30* | 6 | 0 | 0.0 | 4.8 | 0.0 | 29 |
| *TRH.22T25nRH.L40.nighttime.sum_25* | 12 | -10 | 23 | *fa3.12_-10.TRH.22T25nRH.L40.nighttime.sum_25* | 3 | 0 | 0.0 | 3.1 | 0.0 | 23 |
| *T.16T19.nighttime.sum_30* | 5 | -1 | 7 | *fa3.5_-1.T.16T19.nighttime.sum_30* | 0 | 337 | 412.5 | 449.0 | 471.8 | 575 |
| *T.10T13.dawn.sum_30* | -2 | -8 | 7 | *fa3.-2_-8.T.10T13.dawn.sum_30* | 0 | 34 | 228.8 | 247.8 | 290.0 | 336 |

^a^ NA is the number of missing values, min = minimum, q1 = first quartile, q3 = third quartile, and max = maximum values. ‘Count’-based variables (e.g., *RH.90.rl.count6.dusk.sum_20*) and ‘*peak*’ base (e.g., *RH6.peak4.nighttime.sum_20*) measure the number of events during the optimal period, while other variables measure accumulated hours.

^b^ Missing values in descriptive summary at the beginning and end of the season was due to variations in crop growing season length among environments. However, no correlation analyses were conducted only if at least 10 environments provided responses.
